# Prevalence of virulence- and antibiotic resistance-associated genotypes and phenotypes in *Staphylococcus aureus* strains from the food sector compared to clinical and cow mastitis isolates

**DOI:** 10.3389/fcimb.2024.1327131

**Published:** 2024-01-29

**Authors:** Andrea Jurado, Lucía Fernández, Ana Rodríguez, Pilar García

**Affiliations:** ^1^ Department of Technology and Biotechnology, Instituto de Productos Lácteos de Asturias (IPLA-CSIC), Asturias, Spain; ^2^ DairySafe Group. Instituto de Investigación Sanitaria del Principado de Asturias (ISPA), Oviedo, Spain

**Keywords:** bacteriophages, antibiotic resistance, food industry, biofilms, *Staphylococcus aureus*

## Abstract

**Background:**

Infections by the pathogen *Staphylococcus aureus* currently represent one of the most serious threats to human health worldwide, especially due to the production of enterotoxins and the ability to form biofilms. These structures and the acquisition of antibiotic resistance limit the action of antibiotics and disinfectants used to combat this microorganism in the industry and the clinic.

**Methods:**

This work reports a comparative phenotypic and genotypic study of 18 *S. aureus* strains from different origins: clinical samples, milk from mastitic cows and food industry surfaces, most of which were isolated in Northern Spain.

**Results:**

Genetically, the strains were very diverse but, in most cases, a closer proximity was observed for those from the same source. Notably, the average number of virulence genes was not significantly different in strains from the food sector. Of the 18 strains, 10 coded for at least one enterotoxin, and four of them carried 6 or 7 enterotoxin genes. The latter were all veterinary or clinical isolates. Most strains carried prophages, plasmids and/or pathogenicity islands. Regarding antibiotic resistance, although phenotypically all strains showed resistance to at least one antibiotic, resistance genes were only identified in 44.5% of strains, being mastitis isolates those with the lowest prevalence. Virulence-related phenotypic properties such as haemolytic activity, staphyloxanthin production, biofilm-forming capacity and spreading ability were widely distributed amongst the isolates.

**Conclusions:**

Our results indicate that production of virulence factors, antibiotic resistance and biofilm formation can be found in *S. aureus* isolates from diverse environments, including the food industry, although some of these traits are more prevalent in strains isolated from infections in cows or humans. This emphasizes on the importance of monitoring the spread of these determinants not only in samples from the clinical environment, but also along the food chain, a strategy that falls under the prism of a one-health approach.

## 1 Introduction

Foodborne diseases remain one of the most serious health problems worldwide. According to the latest available EFSA report, 4,005 foodborne outbreaks (FBOs) and 32,543 human cases, including 2,495 hospitalizations and 31 deaths, were reported by the EU Member States in 2021 (EFSA and ECDC (European Food Safety Authority and European Centre for Disease Prevention and Control), 2022). These figures represent a notable increase compared to 2020 (between 29-62%). The number of FBOs involving bacterial toxins has also increased by 152 cases in 2021 (EFSA and ECDC (European Food Safety Authority and European Centre for Disease Prevention and Control), 2022). Amongst toxin-derived outbreaks, those produced by coagulase-positive staphylococci, such as *Staphylococcus aureus*, led to the highest hospitalization rates, and are amongst the most common foodborne diseases in many parts of the world (Hennekinne et al., 2012). This microorganism commonly inhabits the bodily surfaces of humans and other animals, most frequently without causing disease. Indeed, about 70-90% of the population is considered to be, at least transiently, asymptomatic carriers of *S. aureus* (Hasse-Cieślińska, 2007). With this in mind, it is not surprising that the main routes of food contamination are workers, during food processing, and farm animals (Argudín et al., 2010). Additionally, dairy animals, such as cows, goats or sheep, can also suffer from staphylococcal infections, especially mastitis, which causes large economic losses in the livestock sector (Ajose et al., 2022). Some of the foods that pose a greater risk of contamination with this bacterium include meat and meat products, poultry and egg products, milk and dairy products, bakery products (cream-filled pastries and cakes) and ready-to-eat foods (Kadariya et al., 2014). Contamination with *S. aureus* frequently takes place during food processing. As a result, the isolation of this microorganism from food contact surfaces in the dairy, meat and seafood industries is highly documented (Gutiérrez et al., 2012), and is considered an indicator of inadequate cooking and/or food processing (Castro et al., 2018).

Apart from food poisoning, *S. aureus* can also cause a wide range of infections in humans, especially when their immune system is compromised, being currently considered one of the most notorious nosocomial pathogens (Kadariya et al., 2014; WHO (World Health Organization), 2021). This ability to cause disease is due to the production of diverse toxins (e.g. enterotoxins, leukotoxins, hemolysins, exfoliative toxin, etc.) and the possession of immune evasion mechanisms (e.g. antiphagocytic capsule, sequestration of host antibodies, antigen masking by protein A, intracellular survival, blocking of leukocyte chemotaxis, etc.) (Hennekinne et al., 2012; Malak et al., 2020).

*S. aureus* enterotoxins are the main cause of food poisoning, as they are able to persist in food even after denaturing treatments that kill bacteria, and are resistant to the action of human enzymes such as pepsin and trypsin (Fisher et al., 2018). As superantigens, their effect leads to the activation of a high number of T cells, causing cytokine release and, sometimes, systemic shock (Fisher et al., 2018). The currently described enterotoxins can be classified into staphylococcal enterotoxins with emetic activity (SEA-SEE, SEG-SEI, SER-SET) and staphylococcal-like enterotoxins, which do not have a demonstrated emetic activity in a primate model or have not been tested yet (SE*l*L, SE*l*Q, SE*l*J, SE*l*K, SE*l*M to SE*l*P, SE*l*U, SE*l*U2 and SE*l*V). They can also be divided into classical (SEA to SEE) and new (SEG to SE*l*U2) enterotoxins, the latter being encoded by the enterotoxin gene cluster (Argudín et al., 2010; Johler et al., 2015).

Genes encoding virulence factors, including the aforementioned enterotoxins, and antibiotic resistance are often located in mobile genetic elements, such as plasmids or bacteriophages, increasing their distribution potential (Ayliffe et al., 1996; Hennekinne et al., 2012). These phages capable of exchanging genes between bacterial cells belong mostly to the so-called temperate phages, and have as a main feature the ability of integrating into the bacterial chromosome as prophages. Based on their role in horizontal gene transfer, temperate phages pose a serious problem regarding the treatment of these strains; hence the importance of their detection and characterization (Goerke et al., 2009). Antibiotic resistance development can also be associated with the acquisition of chromosomal cassettes, transposons located in conjugative plasmids or chromosomal mutations. Worryingly, some *S. aureus* strains have evolved resistance to many of the currently available antibiotics, including methicillin and even vancomycin (McGuinness et al., 2017; Lee et al., 2018). As a result, the WHO has categorized this microbe as a high priority pathogen (WHO (World Health Organization), 2021). Indeed, healthcare-acquired methicillin-resistant *S. aureus* (HA-MRSA) strains constitute a serious problem in the hospital environment, causing serious diseases, such as bacteremia, ventilator-associated pneumonia and surgical wound infections, that often result in therapeutic failure (Kanafani and Fowler, 2006; Pofahl et al., 2011; Hurley, 2018). In addition, the spread of these MRSA strains has extended the problem to the out-of-hospital community (CA-MRSA) (de Kraker et al., 2011). This bacterium is also well known for its ability to persist in hostile environments, including body tissues, by forming biofilms, in which bacterial cells are surrounded by an extracellular matrix consisting of polysaccharides, proteins and/or DNA (Flemming and Wingender, 2010). This complex structure confers biofilms with high resistance to antibiotics, as well as to the cleaning and disinfection systems commonly used in the food industry (Bridier et al., 2015; Olsen, 2015). Staphylococcal biofilms have been associated with endovascular, bone and joint infections, as they can grow and persist on catheters and prostheses. Moreover, biofilms are one of the best ways for this bacterium to survive on food industry surfaces and, ultimately, contaminate foods (Castro et al., 2018).

The determination of genetic markers associated with strains from specific environments or geographical areas can be used to study the dispersion path of a strain or its mobile elements and identify its source. This would allow the implementation of more refined measures to prevent its expansion. Also, the levels of certain virulence factors can help establish the degree of severity of the pathology, as in the case of leukocidin LukM/F, which is associated with zoonotic infections and can be detected in the milk of mastitic cows (Vrieling et al., 2016).

In the present work, we carried out an in depth analysis of several strains from Northern Spain from different sources: the food (meat and dairy) industry and mastitic cows, many of which had been isolated as part of the same study. Additionally, these strains were compared to well-characterized reference strains of clinical origin. Regarding the food and mastitis strains, our most significant finding was that despite having the same geographic origin and, in some cases, having been isolated within a short time period, they are actually quite genetically diverse.

## 2 Materials and methods

### 2.1 Bacterial strains, bacteriophages and culture conditions

Eighteen different *S. aureus* strains from different origins were used in this study (**Table 1**). All the bacteria were routinely cultured in tryptic soy broth (TSB; Scharlau, Barcelona, Spain) at 37°C with shaking or on TSB plates containing 2% (wt/vol) bacteriological agar (TSA).

**Table 1:** Typing and origin of the different *S. aureus* strains used in this study.

Strain	Origin	Typing				Reference
		MLST type	SPA type	Capsule type	Agr type	
SH1000	Derived from clinical strain NCTCT8325 (sepsis patient, USA)	8	t211	5	I	Horsburgh et al., 2002
MW2	Clinical (septic arthritis, USA)	1	t128	8	III	Centers for Disease Control and Prevention (CDC), 1999
132	Clinical (foreign body infection, Navarra, Spain)	8	t008	5	I	Vergara-Irigaray et al., 2009
RN4220	Derived from clinical strain NCTCT8325 (sepsis patient, USA)	8	t211	5	I	Kreiswirth et al., 1983
Newman	Clinical (tubercular osteomielitis, UK)	254	t008	5	I	Duthie and Lorenz, 1952
JE2	Derived from clinical strain USA300 LAC (abscess, USA)	8	t008	5	I	Fey et al., 2013
V329	Mastitis (Navarra, Spain)	126	t605	5	II	Cucarella et al., 2001
Sa9	Mastitis (Asturias, Spain)	504	t529	8	II	García et al., 2007
Sa5	Mastitis (Asturias, Spain)	97	t359	5	I	Unpublished
Sa7	Mastitis (Asturias, Spain)	146	t002	5	II	Unpublished
IPLA19	Mastitis (Asturias, Spain)	unknown	t529	8	II	Gutiérrez et al., 2012
IPLA1	Dairy Industry (Asturias, Spain)	97	t527	5	I	Gutiérrez et al., 2012
IPLA3	Dairy Industry (Asturias, Spain)	398	t011	5	I	Gutiérrez et al., 2012
IPLA5	Dairy Industry (Asturias, Spain)	2826	t359	5	I	Gutiérrez et al., 2012
IPLA11	Meat Industry (Asturias, Spain)	15	t254	8	II	Gutiérrez et al., 2012
IPLA13	Meat Industry (Asturias, Spain)	1460	t156	8	II	Gutiérrez et al., 2012
IPLA15	Meat Industry (Asturias, Spain)	1	t1491	8	III	Gutiérrez et al., 2012
IPLA16	Meat Industry (Asturias, Spain)	12	t213	8	II	Gutiérrez et al., 2012

### 2.2 Genome sequencing, assembly and annotation

The sequences of strains SH1000, MW2, 132, RN4220, Newman, JE2 and V329 were available in public databases with accession numbers JANFOB010000001, NC_003923, NZ_ACOT01000046, NZ_WWFP01000001, NC_009641, NZ_CP020619 and JAGTJH000000000, respectively.

The following *S. aureus* strains were grown on TSA plates at 37 °C: Sa9, Sa5, Sa7, IPLA19, IPLA1, IPLA3, IPLA5, IPLA11, IPLA13, IPLA15, IPLA16. A single colony from each strain was then streaked out on a fresh plate and incubated overnight. All cells grown on this plate were harvested and resuspended in 1 ml PBS to obtain approximately 5 x 10^9^ cells. To estimate the number of cells, dilutions of the resulting suspension were made to obtain an OD_600_ of 0.1, which corresponds to 1 x 10^7^ cells. The final number was calculated by correcting by the dilution factor. Cells were pelleted and washed with PBS and, after centrifuging again at maximum speed for 3 minutes, resuspended in 0.5 ml of 1 x DNA/RNA Shield buffer (Zymo Research, Irvine, USA) until gDNA isolation. Samples were treated with 0.2 mg/ml lysostaphin and 0.1 mg/ml RNase A (ITW Reagents, Spain) for 25 min at 37 °C. These samples were further incubated for 5 min at 65 °C with 0.1 mg/ml proteinase K (VWR Chemicals, Ohio, USA) and 0.5% v/v SDS (Sigma-Aldrich, Missouri, USA). Purification of gDNA was carried out using solid-phase reversible immobilization (SPRI) beads (Beckman Coulter, Brea, USA), and the genomic libraries were prepared according to the manufacturer’s protocol using Nextera XT Library Prep Kit (Illumina, San Diego, USA). The resulting libraries were then sequenced on an Illumina HiSeq platform using a 250 bp paired end protocol. Additionally, strains Sa9, IPLA15 and IPLA16 were sequenced with an Oxford Nanopore sequencing platform to obtain long-read sequencing data. Genome sequencing was provided by MicrobesNG (http://www.microbesng.com). Preparation of the DNA libraries was carried out with Oxford Nanopore SQK-LSK109 kit with Native Barcoding EXP-NBD104/114 (ONT, United Kingdom) using 400-500 ng of high-molecular-weight DNA. Next, the barcoded samples were pooled together into a single sequencing library before being loaded in a FLO-MIN106 (R.9.4.1) or FLO-MIN111 (R10.3) flow cell in a GridION (ONT, United Kingdom).

Unless otherwise noted, genome analysis was carried out using default parameters for all software. Quality of the reads was checked with FASTQC v. 0.11.3 (Andrews, 2010) and trimming was performed with Trimmomatic v. 0.39 by using a sliding window quality cutoff of Q15 (Bolger et al., 2014). *De novo* assembly of the genomes was achieved using SPAdes v. 3.14.1 (Bankevich et al., 2012). In the case of strains Sa9, IPLA15 and IPLA16, hybrid assemblies were generated using Unicycler version 0.4.0 (Wick et al., 2017). Quality of the assemblies was assessed with QUAST v. 5.0.2 (Gurevich et al., 2013). Sequences shorter than 500 bp were removed prior to genome annotation with the NCBI Prokaryotic Genome Annotation Pipeline v. 5.1 (Tatusova et al., 2016) for strains Sa9, IPLA15 and IPLA16 and Prokka (Seemann, 2014) for the rest.

### 2.3 Analysis of mobile genetic elements

The annotated genomes of all the strains were screened for the presence of genomic islands with the tool IslandViewer 4 (IslandViewer 4 (https://www.pathogenomics.sfu.ca/islandviewer) (Bertelli et al., 2017). Identification and annotation of prophage sequences and plasmids was performed by using PHASTER (PHASTER, http://phaster.ca/) (Arndt et al., 2016) and PlasmidFinder 2.1 (https://cge.cbs.dtu.dk/services/PlasmidFinder-2.0/) (Carattoli et al., 2014), respectively. For the analysis of the SCCmec elements, the tool SCCmecFinder1.2 from the same webserver was used with the following parameters: 90% minimum identity and 60% minimum coverage (CGE Server (https://cge.dtu.dk/services/SCCmecFinder) (International Working Group on the Classification of Staphylococcal Cassette Chromosome Elements (IWG-SCC), 2009). This latter analysis was only carried out in *mecA* positive strains.

### 2.4 Phylogenetic analysis

A single nucleotide polymorphism (SNP) tree was constructed using CSI Phylogeny 1.4 (CGE Server (https://cge.food.dtu.dk/services/CSIPhylogeny/) (Kaas et al., 2014) and the assembled FASTA files of all *S. aureus* strains sequenced in this study together with the following reference strains from GenBank: *S. aureus* SH1000 (JANFOB010000001.1), MW2 (NC_003923.1), JE2 (NZ_CP020619.1), 132 (NZ_ACOT01000046.1), RN4220 (NZ_WWFP01000001.1), Newman (NC_009641.1) and V329 (JAGTJH000000000). This analysis was carried out using default settings and excluding heterozygous SNPs. The following criteria for high-quality SNP calling and filtering were chosen: (i) a minimum depth of 10× at SNP positions; (ii) a minimum relative depth of 10% at SNP positions; (iii) a minimum distance of 10 bp between SNPs; (iv) a minimum SNP quality of 30; (v) a minimum read mapping quality of 25; and (vi) a minimum Z score of 1.96.

### 2.5 Typing and identification of virulence and antimicrobial resistance genes

The presence of antimicrobial resistance and virulence genes was evaluated by using two resources, namely, ResFinder4.1 (https://cge.cbs.dtu.dk/services/ResFinder/) (Bortolaia et al., 2020) and VFDB (VFDB: Virulence Factor Database (http://www.mgc.ac.cn/cgi-bin/VFs/v5/main.cgi) (Liu et al., 2019), respectively, using the default parameters. This last tool was also used to carry out a comparative analysis of our strain collection with *S. aureus* strains from different origins and geographical locations (**Table S3**). The presence of phenol-soluble-modulin genes (PSMs-α, PSM-β, *hld*) and mutations in selected genes of interest was analyzed with BLAST https://blast.ncbi.nlm.nih.gov/Blast.cgi) (Camacho et al., 2009). To determine the sequence type (ST) of the isolated strains, *in silico* MLST analysis was performed using the MLST 2.0 database (MLST 2.0 (https://cge.cbs.dtu.dk/services/MLST/) (Larsen et al., 2012). The *S. aureus* MLST scheme consisted of 7 housekeeping genes: *arcC* (carbamate kinase), *aroE* (shikimate dehydrogenase), *glpF* (glycerol kinase), *gmk* (guanylate kinase), *pta* (phosphate acetyltransferase), *tpi* (triosephosphate isomerase), and *yqi* (acetyl coenzyme A acetyltransferase). The parameter 5X for minimum depth for an allele was selected and the assembled genomes were used as input.

Spa types were predicted using spaTyper v1.0 webserver from the Center of Genomic Epidemiology (https://cge.cbs.dtu.dk/services/spatyper) (Bartels et al., 2014). In this typing technique, 21 to 27 polymorphic VNTR in the 3-coding region of staphylococcal protein A (spa) were compared in order to assign a unique repeat code corresponding to its spa type.

### 2.6 Biofilm formation by different bacterial strains

Biofilm assays were carried out as described previously, with some modifications (Herrera et al., 2007). Briefly, overnight cultures of different *S. aureus* strains were diluted 100-fold in fresh TSBg (TSB supplemented with 0.25% w/v D-(+)-glucose) and 200 μl-aliquots were poured into each well of a 96-well plate (Thermo Scientific, NUNC, Madrid, Spain). Biofilms were grown for 24 h at 37 °C and, subsequently stained with 0.1% crystal violet. To do that, the planktonic phase was removed and the adhered cells were washed twice with PBS (137 mM NaCl, 2.7 mM KCl, 10 mM Na_2_HPO_4_ and 2 mM KH_2_PO_4_; pH 7.4). Afterwards, 200 μl of crystal violet were added and, following incubation for 15 minutes at room temperature, excess of the dye was removed by washing the biofilms with water. Finally, the crystal violet retained in the biofilm was solubilized with 33% (v/v) acetic acid, and the total biomass was quantified by measuring absorbance at 595 nm (A_595_) with a Bio-Rad Benchmark plus microplate spectrophotometer (Bio-Rad Laboratories, Hercules, CA, USA).

### 2.7 Hemolysin, staphyloxanthin and protease production

To determine staphyloxanthin production, 5 μl from an overnight culture of each strain were placed on a TSA plate, which was then incubated for 20-24 hours at 37 °C. The next day, staphyloxanthin production was determined by visual inspection as the presence of an orange-yellow color. On the other hand, for the characterization of hemolysin production, 5 μl from an overnight culture of each strain were laid on a blood agar plate and after 24 hours of incubation at 37 °C, the action of alpha hemolysin was detected by visual analysis as the presence or absence of a halo surrounding the colonies. The plates were observed again after another 24 hours of incubation at 4 °C in order to observe the effect of the beta hemolysin (Smyth et al., 1975). The detection of protease activity was carried out on plates containing nutrient broth (Becton, Dickinson and Company, Sparks, USA), supplemented with 1.5% agar and 1.5% skim milk (OXOID, Hampshire, England). These plates were inoculated with 5 μl from *S. aureus* overnight cultures and subsequently incubated for 24 h at 37 °C. After incubation, the proteolytic activity was considered positive when a clear zone was observed around the bacterial colonies.

### 2.8 Colony spreading ability of different strains

Fresh culture plates were prepared with autoclaved TSB medium containing 0.25% agar. Once dried, a volume of 2 μl from an overnight culture of each strain was inoculated on the center of each plate and incubated overnight at 37°C. After incubation, the diameter of each bacterial colony was measured.

### 2.9 Antimicrobial susceptibility test

The antimicrobial susceptibility profile of the *S. aureus* isolates was determined by the Kirby–Bauer disk diffusion method (Biemer, 1973). Briefly, overnight cultures of the different strains were diluted 10-fold in PBS and the suspension was streaked on 20-ml TSA plates and allowed to dry. Antibiotic disks were then placed on each plate with a considerable separation between them and incubated at 37°C for 24 h. After the incubation time, the zones of inhibition were measured with a ruler and interpreted in order to compare the antimicrobial susceptibility of the strains. The following antimicrobial agents were used: ampicillin (10 µg), methicillin (10 µg), tetracycline (30 µg), chloramphenicol (30 µg), gentamicin (10 µg), tobramycin (10 µg), erythromycin (15 µg), ciprofloxacin (5 µg), vancomicin (5 µg), streptomycin (10 µg). Antibiotic disks were purchased from Oxoid (Wade Road, Basingstoke, Hants, UK).

### 2.10 Statistical analysis

All experiments were performed in triplicate. Different groups of samples were compared by using one-way analysis of variance (ANOVA) followed by a *post hoc* Tukey HDS test. P-values <0.05 were considered statistically significant.

### 2.11 Genome accession numbers

The genome assemblies of 11 *S. aureus* strains were deposited in the DDBJ/ENA/GenBank database under the following accession numbers: CP134618-CP134619 (IPLA15), CP134617 (IPLA16), CP134620 (Sa9), JAVRXU000000000 (IPLA1), JAVRXT000000000 (IPLA3), JAVRXS000000000 (IPLA5), JAVRXR000000000 (IPLA11), JAVRXQ000000000 (IPLA13), JAVRXV000000000 (IPLA19), JAVRXX000000000 (Sa5) and JAVRXW000000000 (Sa7).

## 3 Results

### 3.1 Genomic analysis of *S. aureus* strains

The aim of this study was the genomic and phenotypic comparison of 18 *S. aureus* strains from different origins, including 5 from cows with mastitis (V329, Sa9, Sa5, Sa7, IPLA19), 4 from the meat industry (IPLA11, IPLA13, IPLA15, IPLA16), 3 from the dairy industry (IPLA1, IPLA3, IPLA5), 4 from the clinic (JE2, MW2, 132, Newman) and 2 laboratory strains (SH1000 and RN4220) derived from the clinical strain *S. aureus* NCTC 8325 (**Table 1**). In order to do that, we sequenced the genomes of 11 strains, namely Sa9, Sa5, Sa7, IPLA19, IPLA1, IPLA3, IPLA5, IPLA11, IPLA13, IPLA15, IPLA16, whose genomes were not available in public databases. **Supplementary Table 1** summarizes the results regarding genome assembly and annotation. The genome size ranged from 2,708,529 to 2,805,322 bp, and the G+C content was very similar between the strains, with 32.70% and 32.87% being the minimum and the highest values, respectively.

**Figure 1:**
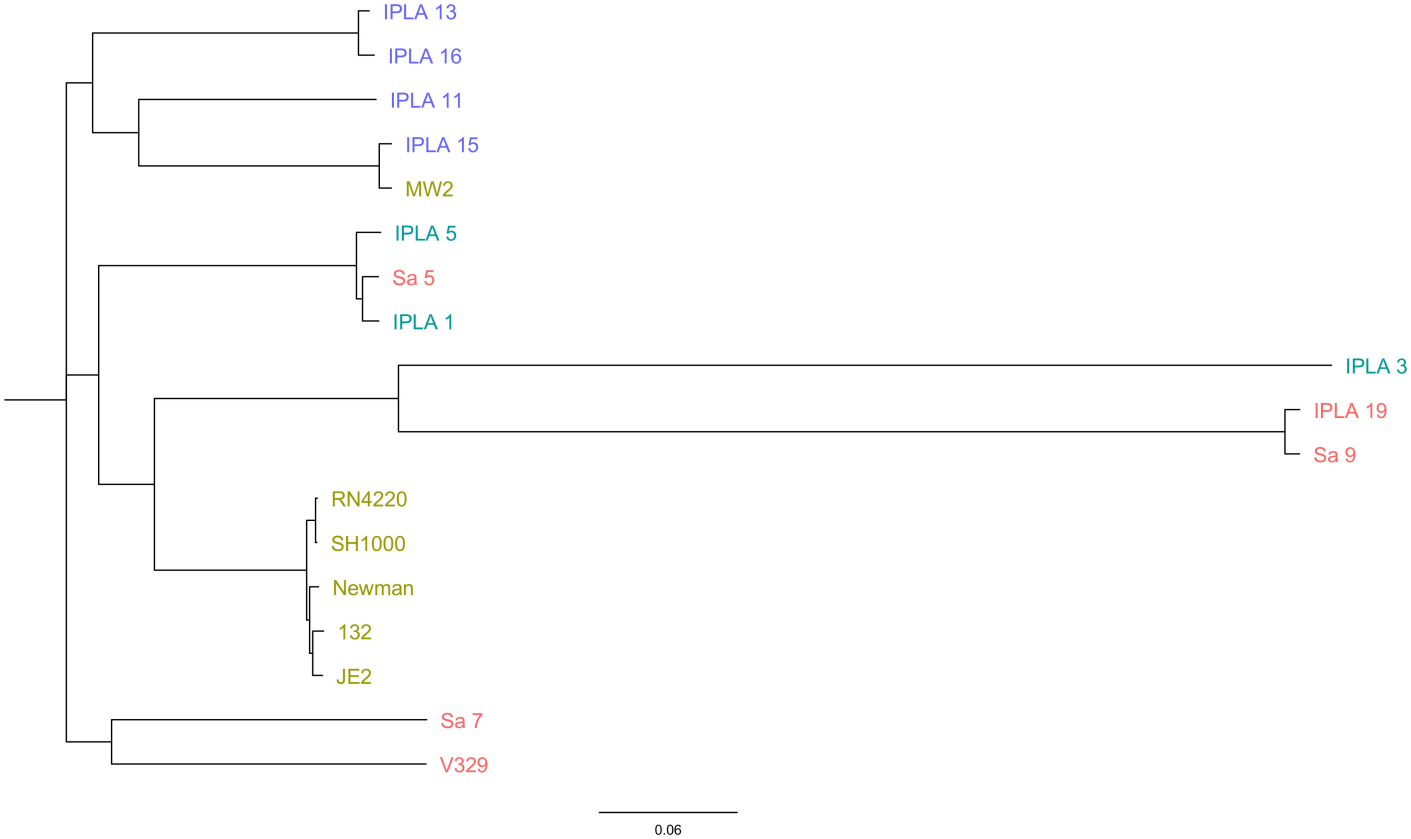
Phylogenetic analysis of *S. aureus* isolates. The genome-wide SNP-based maximum likelihood phylogenetic tree of *S. aureus* isolates obtained from different origins (Clinical sector: Green, Food industry: Purple (meat industry), Blue (Dairy Industry), mastitis: Red). The genome of strain NCTC 8325 was used as the reference to map and screen the SNPs. The phylogenetic tree was constructed using the CSI Phylogeny v1.4 and visualized using FigTree.

Once all the genome sequences were available, we carried out a comparative genomic analysis of the 18 strains. All of them showed high diversity regarding their multilocus sequence typing (MLST) profile (**Table 1**). A similar trend was observed for the spa types, which were also very diverse. Only one type (t008) was found in several clinical strains (JE2, Newman, 132) (**Table 1**). Regarding capsule type, 39% (7/18) of the strains had capsule type 8, while 61% (11/18) were type 5 (**Table 1**). It is noteworthy that all the strains from the meat industry were type 8, whereas those from the dairy industry were type 5. Amongst the strains of clinical origin, most were type 5 except for MW2, which had capsule type 8, and those from mastitic cows presented in similar numbers capsule type 5 (V329, Sa5, Sa7) and 8 (Sa9, IPLA19). The most prevalent agr type was type I (50% of the strains), followed by type II (39%) and type III (11%) (**Table 1**). Again, MW2 (agr type III) was the only clinical strain that did not have agr type I. Also, strains from the dairy industry all were type I, whereas most strains from mastitis and the meat industry were type II except for Sa5 (type I) and IPLA15 (type III). The phylogenetic tree obtained based on SNP analysis revealed close proximity between all the clinical strains, including the laboratory strains, with the exception of MW2, which clustered with the meat industry isolates (**Figure 1**). Some mastitis isolates (Sa5, Sa9 and IPLA19) grouped fairly close to those from the dairy industry (IPLA5, IPLA1, IPLA3) and in close proximity to the clinical isolates. In contrast, the other two strains from milk of cows with mastitis (Sa7 and V329) formed a separate cluster.

### 3.2 Identification of virulence and antibiotic resistance genes

**Figure 2:**
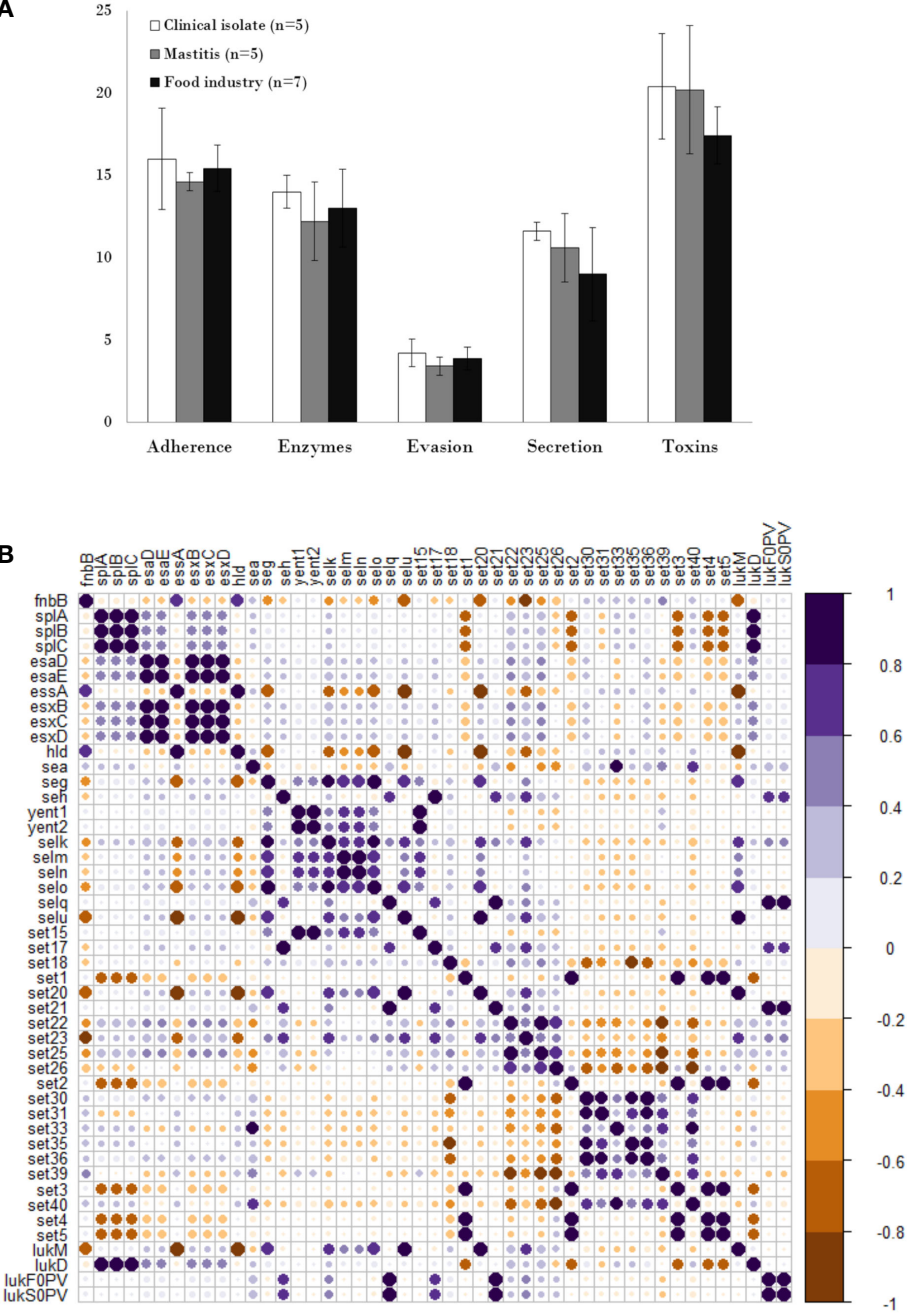
Distribution and pairwise associations of virulence genes in *S. aureus* isolates. **(A)** Distribution of genes encoding different types of virulence factors in *S. aureus* isolated from the clinical sector (white), food industry (black), and mastitis (grey). Data represents the average and standard deviation. **(B)** Pairwise associations of selected virulence genes, computed using phi coefficients (cut-off 0.7), with a colour gradient representing the type of association. The intensity of the colour and the size of the circle indicate the strength of the association.

The presence of different types of virulence factors in 17 of the strains was examined and the results are summarized in **Supplementary Table 2**. Strain SH1000 was not included in this analysis as it is practically identical to RN4220.The virulence factors with the highest prevalence in all isolates regardless of origin were toxin-coding genes (19.11%), followed by adhesion-related genes (15.35%), while those involved in evasion showed the lowest prevalence (3.82%) (**Figure 2A**). In terms of origin, strains of clinical origin exhibited the highest average prevalence of virulence factors of all types (adherence: 16%, enzymes: 14%, evasion factors: 4.2%, secretion: 11.6%, toxins: 20.4%) (**Figure 2A**). Mastitis isolates possessed a higher mean number of genes involved in secretion (10.60%) and toxin production (20.2%) than food isolates, but a lower prevalence of those related to adherence (14.60%), enzymes (12.2%) and evasion (3.4%) (**Figure 2A**). Nonetheless, it must be noted that none of these differences were statistically significant, indicating that food industry strains, exhibited an average number of virulence determinants similar to that of strains from infected cows or humans.

Multiple virulence genes were present in all strains, including adhesion factors (*atl*, *ebp*, *eap*, *efb*, *fnbA*, *icaA*, *icaC*, *icaR* and *spa*), enzymes (*sspB, sspC, sspA, hysA, geh, lip, coa, nuc* and *aur*), evasion factors (*adsA, sbi*, and *isd* family genes), and toxins (*hly/hla, hlgA, hlgB*, and *hlgC*). However, other virulence genes were only observed in a few isolates. These included the enzyme-encoding gene *sak* (JE2, MW2, 132 and Newman from clinical origin; Sa7 from mastitis and IPLA13 from the meat industry) and the evasion factor gene *chp* (JE2 and Newman from clinical origin and IPLA11 from the meat industry). Strains of meat origin showed the lowest possession of type VII secretion genes (*esxB, esxC, esxD, esaA* and *esaE*), which were only present in strain IPLA15. On the other hand, the exfoliative toxin genes were not detected in any strain. It is worth highlighting that the Panton-Valentine leukocidin genes *lukF0PV* and *lukS0PV* were only present in two strains of clinical origin (JE2 and MW2), and the gene coding for the toxic shock syndrome toxin (*tsst*) was only present in the isolate strain Sa9, which together with IPLA19 are the only strains carrying the *lukM* gene. Another interesting finding was that only clinical strains were positive for phenol soluble modulins type α (PSM-α), whereas PSM-β modulins type I and II were present in all strains in the collection. Regarding enterotoxin genes, 55.5% (10/18) of the strains carried at least one gene, with *sea* (27.7%), *sec* (27.7%) and *selk* (27.7%) being the most prevalent (**Table 2**). Four strains possessed 6 or 7 enterotoxin genes, of which three were isolated from mastitic cows and one was a clinical strain. By contrast, seven strains did not carry any enterotoxin genes, four of which were isolated from food industry samples. Some enterotoxin genes (*seb, sed, see, sei, sej, selp* and *selr*) were absent from all strains, while enterotoxin-like genes were more prevalent in mastitis isolates (**Table 2**). With the aim of determining if these trends could be extrapolated to other studies, we examined the presence of different enterotoxins in the genomes of 75 strains from different origins (**Supplementary Table 3**). Just like in our study, no strain carried enterotoxins *sed*, *see*, *sej*, *selp* or *selr*, and none of the mastitis isolates produced *sea* or *seb*. However, in this case, enterotoxin-like genes were not more prevalent in mastitis isolates and were also very frequent in clinical and even in food strains. Another significant difference is that the strains with the highest number of enterotoxin genes were actually food isolates.

**Table 2:** Presence of enterotoxin genes in *S. aureus* isolates.

Strains	Enterotoxin-Classical		Enterotoxin- New										
	*sea*	*sec*	*seg*	*seh*	*selk*	*sell*	*selm*	*seln*	*selo*	*selq*	*selu*	*Yent-1*	*Yent-2*
JE2	–	–	–	–	+	–	–	–	–	+	–	–	–
MW2	+	+	–	+	+	+	–	–	–	+	–	–	–
132	+	–	–	–	–	–	–	–	–	–	–	–	–
RN4220	–	–	–	–	–	–	–	–	–	–	–	–	–
NEWMAN	+	–	–	–	–	–	–	–	–	–	–	–	–
V329	–	–	–	–	–	–	–	–	–	–	–	–	–
Sa9	–	+	+	–	+	+	–	–	+	–	+	–	–
Sa5	–	–	–	–	–	–	–	–	–	–	–	–	–
Sa7	–	–	+	–	+	–	+	+	+	–	–	+	+
IPLA19	–	+	+	–	+	–	+	+	+	–	+	–	–
IPLA1	–	–	–	–	–	–	–	–	–	–	–	–	–
IPLA3	–	–	–	–	–	–	–	–	–	–	–	–	–
IPLA5	–	–	–	–	–	–	–	–	–	–	–	–	–
IPLA11	–	–	–	–	–	–	–	–	–	–	–	–	–
IPLA13	+	+	–	–	–	–	–	–	–	–	–	–	–
IPLA15	–	–	–	+	–	–	–	–	–	–	–	–	–
IPLA16	+	+	–	–	–	–	–	–	–	–	–	–	–
	5/18	5/18	3/18	2/18	5/18	2/18	2/18	2/18	3/18	2/18	2/18	1/18	1/18

Presence (+) or absence (-) of 20 enterotoxin genes. The genes seb, sed, see, sei, sej, selp and selr are not shown because of their absence in all strains.

Analysis of the correlation between the different types of virulence genes in the eighteen strains showed that the adhesion gene *fnbB* exhibited a negative association with genes coding for serine proteases (*splA*, *splB*, *splC*) and type VII secretion systems, except *essA*. In turn, genes coding for serine proteases showed a strong positive association among themselves and with the leukocidin *lukD* gene. Most type VII secretion system genes (*esaD*, *esaE*, *esxB*, *esxC*, *esxD*) showed a pattern of positive association with each other. In contrast, *essA* exhibited a negative association with the other secretion genes and a positive correlation with the presence of toxin *hld.* Regarding enterotoxin genes, many gene pairs (i.e *selm-seln, yent1-yent2, selo-selu, seg-selo, selu-set20*) and groups of three (i.e *set3-set4-set5, set22-set23-set25, set30-set35-set36*) showed a strong positive association between them and a similar pattern of association with genes of the other categories. Regarding leukocidin genes, *lukF0PV* and *lukS0PV* showed a strong positive association with each other and with the *selq, set21* toxins genes, whereas *lukM* is positively associated with the toxin genes *selU* and *set20* (**Figure 2B**).

Regarding antibiotic resistance determinants, we found that 47% (8/17) of all strains presented genes that might lead to resistance to at least one class of antibiotics. However, only two of them (132 and IPLA3) showed a multiresistance profile (resistance to three or more classes of antibiotics). In addition, 53% (9/17) did not carry any detectable antibiotic resistance genes in their genome, being the strains from mastitis the ones that showed the lowest prevalence of such genes (**Table 3**). Genes related to beta-lactam resistance were a common denominator in all strains with resistance genes, being in five strains (IPLA15, IPLA11, IPLA5, IPLA3, Sa7) due to the *blaZ* gene and in three (132, JE2, MW2) due to *mecA*, genes that cause decreased susceptibility to penicillin and methicillin, respectively. Strain IPLA3 possessed both genes. Presence of the staphylococcal cassette chromosome was observed in all *mecA*-positive strains. On the other hand, potential resistance to quinolones was also identified in two strains of clinical origin (JE2 and 132), which had mutations in the genes *gyrA* (p.S84L) and *grlA* (p.S80Y), to aminoglycosides in three strains from different origins (132, Sa7, IPLA3) mediated by the genes *ant(4’)-Ia*, *str*, *aph(3’)-Ia*, and finally to tetracycline and streptomycin only in IPLA3 strain due to the presence of the genes *tetM*, *tetK* and *str*, respectively. It is noteworthy that the *bleO* gene was found in strain 132, which confers resistance to bleomycin, an antibiotic that causes DNA strand breaks (**Table 3**).

**Table 3:** Antimicrobial resistance genes of *S. aureus* isolates.

Strain	Antibiotic Resistance Genotype			Antibiotic Resistance Phenotype										
	Class^a^	Genes^a^	Staphylococcal cassette chromosome^b^	MET^1^	AMP^2^	VA^1^	CIP^2^	STR^1^	TET^2^	E^2^	GEN^2^	TOB^2^	LZD^2^	CL^3^
**JE2**	Quinolones β-lactams	*gyrA, grlA* *mecA*	SCCmec_type_IVa	R	S	S	R	S	S	S	R	R	S	S
**SH1000**	-	*-*	-	S	S	S	I	S	S	S	R	S	S	S
**MW2**	β-lactams	*mecA*	SCCmec_type_IVa	R	R	S	I	S	S	S	R	R	S	S
**132**	Aminoglycosides Glycopeptides β-lactams Quinolones	*ant(4’)-Ia* *bleO* *mecA* *gyrA, grlA*	SCCmec_type_IVc	R	S	S	R	S	S	S	R	R	S	S
**RN4220**	-	*-*	-	S	S	S	I	S	R	S	R	R	S	S
**Newman**	-	*-*	-	S	S	S	I	S	S	S	R	R	S	S
**V329**	-	*-*	-	S	S	S	I	S	S	S	R	R	S	S
**Sa9**	-	*-*	-	S	S	S	I	S	S	S	R	R	S	S
**Sa5**	-	*-*	-	S	S	S	I	S	S	S	R	R	S	S
**Sa7**	β-lactams Aminoglycosides	*blaZ* *aph(3’)Ia*	-	S	R	S	I	S	S	S	R	R	S	S
**IPLA19**	-	*-*	-	S	S	S	I	S	S	S	S	S	S	S
**IPLA1**	-	*-*	-	S	S	S	I	S	R	S	R	R	S	S
**IPLA3**	β-lactams Tetracycline Aminoglycosides	*mecA, blaZ* *tetM, tetK* *aph(6)-Ic (str)*	SCCmec_type_Vc	R	R	S	I	R	R	S	R	R	S	S
**IPLA5**	β-lactams	*blaZ*		S	S	S	I	S	R	S	S	S	S	S
**IPLA11**	β-lactams	*blaZ*		S	R	S	I	S	S	S	S	S	S	S
**IPLA13**	-	*-*		S	S	S	I	S	S	S	S	S	S	S
**IPLA15**	β-lactams	*blaZ*		R	R	S	I	S	S	S	R	R	S	S
**IPLA16**	-	*-*		S	S	S	I	S	S	S	S	S	S	S

^a^β-lactams (blaZ), amoxicillin; amoxicillin + clavulanic acid; ampicillin; ampicillin + clavulanic acid; cefepime; cefixime; cefotaxime; cefoxitin; ceftazidime; ertapenem; imipenem; meropenem; piperacillin; piperacillin + tazobactam; β-lactams (mecA), methicillin; Aminoglycosides (aph(3’)Ia),neomycin; kanamycin; lividomycin; paromomycin; ribostamycin; gentamicin; Aminoglycosides (ant(4’)-Ia), amikacin/tobramycin/bleomycin; Aminoglycosides (aph(6)-Ic), streptomycin; Quinolones, ciprofloxacin; Tetracyclines, doxycycline; tetracycline; minocycline; Glycopeptides (bleO), bleomycin. Resistance cut-off points, ^1^Self-established; ^2^EUCAST breakpoints; ^3^CLSI breakpoints. MET, Methicillin; AMP, Ampicillin; VA, Vancomycin; CIP, Ciprofloxacin; STR, Streptomycin; TET, Tetraclycline; E, Erythromycin; GEN, Gentamicin; TOB, Tobramycin; LZD, Linezolid; CL, Chloramphenicol.

### 3.3 High abundance of pathogenicity islands and other mobile elements in *S. aureus* strains

The prevalence of intact phages among the different groups of strains according to their origin was very homogeneous. Only 28% (5/18) of the strains did not carry any intact prophages in their genomes, being some strains of clinical origin the ones that showed the highest number (Newman: 4, 132: 3). The % GC of the intact prophages detected ranged between 31.99% and 35.09% (**Table 4**). All strains had at least one pathogenicity island in their genomes, the number being variable among the different strains. Of note, the dairy industry strain IPLA3 possessed the highest number (13), followed by the clinical strain MW2, which had 8 (**Table 4**). The presence of plasmids was also detected in several strains, although 7 did not carry any predicted replicon according to PlasmidFinder (**Table 4**). Some plasmids could be identified by homology with the available databases, and their sizes ranged from 4 to 36 kb. It is worth noting that some of the resistance determinants described above were located in the identified plasmids.

**Table 4:** Genomic analysis of mobile genetic elements.

Strain	Mobile Genetic Elements		
	Phage^a^	Plasmid^b^	Pathogenicity Island
JE2	2	rep7c	7
SH1000	–	–	–
MW2	1	–	8
132	3	rep22	6
RN4220	–	rep7c	2
Newman	4	rep7c	7
V329	2	–	4
Sa9	–	–	5
Sa5	1	–	5
Sa7	1	Rep20	5
IPLA19	1	–	6
IPLA1	1	–	4
IPLA3	1	rep7a repUS43	13
IPLA5	–	repUS5	5
IPLA11	1	rep5a rep16	3
IPLA13	2	–	1
IPLA15	1	rep5a rep7c rep16	5
IPLA16	–	–	4

a Number of intact prophage sequences identified by PHASTER.

b Replicons identified by PlasmidFinder.

### 3.4 Phenotypic analysis of virulence properties

**Figure 3:**
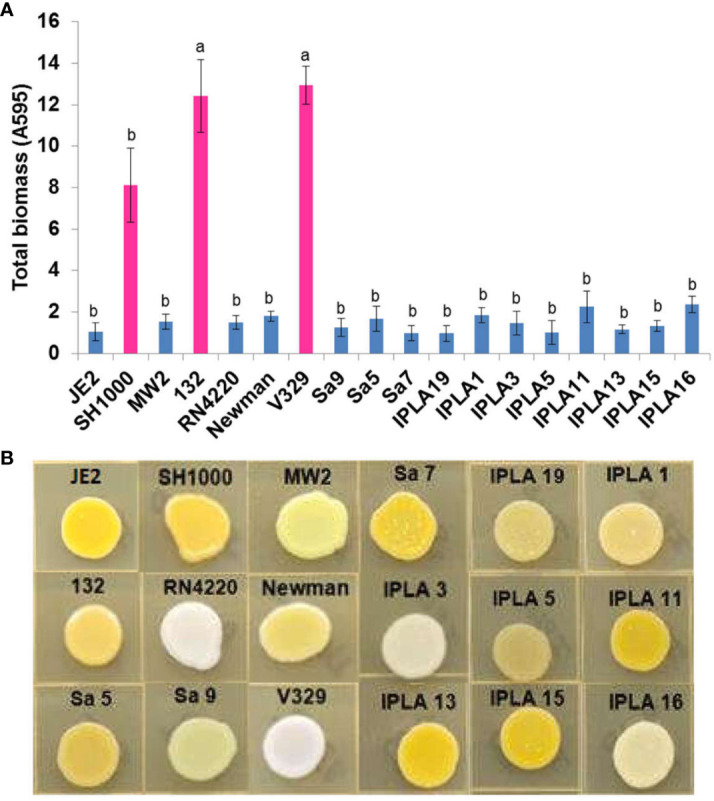
Biofilm and staphyloxantin production of *S. aureus* isolates. **(A)** Biofilm production of 17 *S. aureus* strains from different origin (Clinical sector, Food industry, Mastitis) by conventional crystal violet staining technique. Bars represent mean ± standard deviation of three biological replicates of each strain. Bars within each strain having distinct lower case letter indicate that the biofilm production is statistically different (p < 0.05). Pink colour represents the most biofilm-producing strains. **(B)** Staphyloxantin production of 18 *S. aureus* strains from different origin.

In addition to genomic characterization, we also studied several phenotypes of interest in the different isolates, namely biofilm formation, hemolysin activity, staphyloxanthin production, colony spreading, proteolytic activity and antibiotic resistance. Regarding biofilm formation, all strains displayed some degree of adherence to the surface but only strains V329, SH1000 and 132 were strong biofilm formers (**Figure 3A**). These three strains were a mastitis isolate, a laboratory strain of clinical origin, and a clinical isolate, respectively. Two of them presented exclusive genes like *bap* in V329 and *sasG* in Newman. They also lacked the *icaD* gene, belonging to the staphylococcal *icaADBC* operon involved in the production of PIA/PNAG (polysaccharide intercellular adhesin). In the case of strain 132 the quorum sensing gene *agrC* was missing. Also noteworthy was the lack of *icaB* in the IPLA11 strain. Additionally, the genes *clfA, clfB*, related to the clumping mechanism, were found in some strains (i.e SH1000, JE2, MW2, RN4220, Newman, Sa9, IPLA5) (**Supplementary Table 2**).

A greater diversity was found in terms of staphyloxanthin production. This carotenoid pigment has antioxidant properties and helps the microbe evade the action of reactive oxygen species produced by the immune system (Clauditz et al., 2006). Two strains (V329, RN4220) did not produce any pigment, one of them (Sa9) only synthesized an intermediary pigment and the rest produced varying levels of staphyloxanthin. The absence of production in the above mentioned strains could be explained because of the inactive SigB caused by a deletion in the *rsbU* gene in RN4220 and a mutation in *crtN* in V329, a gene that encodes a dehydrosqualene desaturase which dehydrogenates dehydrosqualene (the first compound in the staphyloxanthin biosynthesis pathway) (Pelz et al., 2005). Also, in strain Sa9 the light yellow production is likely caused by a mutation in gene *crtP*, whose product is responsible for the oxidation of the terminal methyl group of 4,4′-diaponeurosporene to form 4,4′-diaponeurosporenic acid (Pelz et al., 2005) (**Figure 3B**).

**Figure 4:**
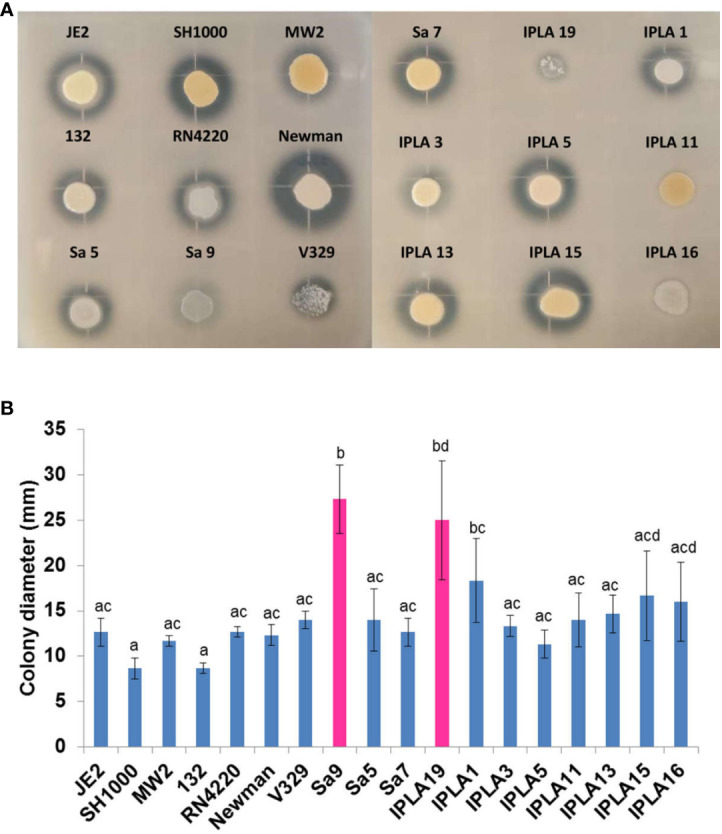
Protease production and spreading capacity of *S. aureus* isolates. **(A)** Protease production of 18 *S. aureus* strains from different origin. **(B)** Expansion of 18 *S. aureus* strains from different origin. Bars represent mean ± standard deviation of three biological replicates of each strain. Bars within each strain having distinct lower case letter indicate that the biofilm production is statistically different (p < 0.05). Pink colour represents the strains with the largest displacement diameter (mm).

All but two strains (132, IPLA11) displayed haemolytic activity on blood agar. This is consistent with the presence in all strains of the *hla* (alpha-hemolysin) and *hlb* (beta-hemolysin) genes except in strains JE2 and 132, which lacked an intact *hlb*. In the case of strain 132, the absence of haemolytic activity is likely due to a mutation in *agrC*, which might prevent expression of the quorum-sensing-dependent gene *hla* gene (Le and Otto, 2015). We could not pinpoint the mutation(s) behind the negative phenotype of strain IPLA11.The highest proteolytic activity was observed in the clinical strains SH1000 and Newman, and the food industry isolates IPLA5, IPLA13 and IPLA15. In contrast, strains Sa9 and IPLA19 (mastitis) together with IPLA11 and IPLA16 (meat industry) showed very low or no protease production, respectively (**Figure 4A**). Proteases allow *Staphylococcus* to degrade host tissue or food components (in the case of industry isolates) to obtain nutrients (Dinges et al., 2000). All strains showed some degree of colony spreading, as can be observed in **Figure 4B**. This phenotype provides an indirect measure of the production of PSMs, which are known virulence factors. The strains with the largest colony diameter were both from mastitis, namely Sa9 (27.33 mm) and IPLA19 (25 mm). These two strains showed a mutation in gene *hld* that may lead to the production of a truncated protein, which has been demonstrated to cause overproduction of PSMs. Mutations in *hld* were also found in 20% of the mastitis isolates included in **Supplementary Table 3**, as well as in 8% of strains from the food industry. In contrast, the strains with the smallest diameter both belonged to the clinical sector, SH1000 (8.67 mm) and 132 (8.67 mm); this latter strain had a mutation in the quorum-sensing gene *agrC*.

### 3.5 Antibiotic resistance

All strains displayed total (R) o partial resistance (I), although some were only resistant to one (16.7%, 3/18) or two (16.6%, 3/18) antibiotics, while others exhibited a multiresistance profile, being resistant to three (22.2%, 4/18), four (27.8%, 5/18), five (11.1%, 2/18) or even seven (1/18, IPLA3) antibiotics. The antibiotics against which we found the highest number of resistant strains were gentamicin (13 strains) and tobramycin (12 strains), followed by ciprofloxacin to which all strains showed intermediate or total resistance (**Table 3**). It is worth noting that all mastitis isolates were susceptible to methicillin. IPLA3 was the only tetracycline resistant strain. In most cases, these phenotypes were in agreement with the genotyping analysis (**Table 3**), although there were some exceptions. For instance, strain IPLA15 was methicillin resistant despite lacking gene *mecA*, and some tetracycline-resistant strains did not carry any specific genetic determinant.

## 4 Discussion

*S. aureus* is a ubiquitous microbe that can cause infections in different animals and contaminate the food environment, potentially leading to food poisoning. As strains spread from one environment to another, they carry their antibiotic resistance and virulence determinants. However, adaptation to the new milieu will involve changes in the bacterial genome due to mutations and genetic exchanges with neighboring bacteria. In this context, it is important to determine how isolates from diverse sources differ from each other and identify genetic signatures that characterize a given environmental niche. This is especially significant from the perspective of food safety. Strains isolated from human patients typically produce virulence factors and exhibit resistance to antibiotics commonly used in therapy. However, if these same traits are shared by isolates spread along the food chain this would pose a serious danger to the general public. Here, several strains from food surfaces and from cows with mastitis were analyzed and compared to reference strains of clinical origin.

*In silico* genotyping revealed that the studied strains are highly diverse, although some patterns can be observed. The most frequent MLST type is ST8, which appears only in several strains of clinical origin. This type was also one of the most frequent in hospital MRSA and MSSA strains in a genomic sequencing study carried out by (Khan et al. (2022a; 2022b). After that, the most common genotypes are ST97 and ST1. ST97 is the most frequent genotype involved in bovine mastitis, but rare in human infections. However, Manara et al. (2018) identified this MLST type in clinical strains from an Italian paediatric hospital. In turn, ST1 has been associated with MRSA community-acquired infections (Yamamoto et al., 2013). The predominant spa type in this work was t008, as was the case for strains of the same origin in previous studies (Khan et al., 2022a; Khan et al., 2022b). This type is frequently associated with ST8 and SCCmec type IV (Manara et al., 2018), as observed for strains JE2 and 132. Other frequent genotypes, t359 and t529, were found in mastitis isolates. T529 was also prevalent in previous studies concerning *S. aureus* presence in raw milk such as Oliveira et al. (2022). Regarding capsule type, all strains were CP5 or CP8, often referred to as microencapsulated nonmucoid strains, which are typical in both clinical and animal strains (O'Riordan and Lee, 2004; Salimena et al., 2016). The most prevalent *agr* type was type I, followed by type II and type III. Type I was also the most abundant in Chinese *S. aureus* strains of food and human origin (Wang et al., 2021). These source-dependent grouping based on genotyping is also reflected in the phylogenetic tree obtained by SNP analysis.

Clinical strains possess, on average, a larger number of virulence genes than isolates from other origins. In the case of toxins, mastitis strains also have a higher number of determinants than food industry strains. Even though these differences are not statistically significant, they do correlate with the fact that these strains were successful in causing disease. Given the importance of enterotoxins in staphylococcal food poisoning, we looked more closely at the prevalence of enterotoxin-coding genes. Notably, one human and three mastitis isolates have six or seven enterotoxin genes. Mastitis is known to be often related to enterotoxin production (Rall et al., 2014). The high prevalence of enterotoxins in the milk of mastitic cows highlights the importance of controlling this disease in dairy cows and preventing the use of contaminated milk down the dairy chain. It is worth noting that one of the two most frequent enterotoxins involved in staphylococcal foodborne diseases, SEB, is not present in any of the strains studied here (Pinchuk et al., 2010). SEC, the most common enterotoxin involved in bovine mastitis, is present in only two out of five mastitis isolates (Fang et al., 2019). In our work, mastitis isolates had a greater prevalence of non-classical enterotoxin genes than strains from other origins. However, we did not observe this trend in strains from other studies (**Supplementary Table 3**).

In terms of antibiotic resistance determinants, 6 strains from our collection as well as four of the reference strains have no detectable resistance markers in their genomes. Additionally, none of the strains analyzed in this work display resistance to vancomycin, erythromycin, linezolid or chloramphenicol. In the case of erythromycin, this differs from other studies on strains from food and clinical settings where the prevalence was high (Fernandes et al., 2022; Li et al., 2022). In contrast, the results obtained for linezolid and chloramphenicol are similar to those reported for *S. aureus* from human and food origin (Wang et al., 2021) and milk (Oliveira et al., 2022) where resistance to these compounds was absent or low. This is important as linezolid is a new bacteriostatic antimicrobial agent with activity against drug-resistant staphylococci, including VISA and VRSA strains (Lowy, 2003). We observed that resistance to beta-lactams is the most prevalent, in some cases due to *blaZ* and, in others, to *mecA*, the gene responsible for methicillin resistance. Only isolate (IPLA3) possessed the latter gene, but IPLA15 was also resistant to this antibiotic. This phenomenon is known as borderline resistance, and is explained in the literature as the result of beta-lactamase overproduction and/or modification of PBP genes (Chambers, 1997). Since IPLA15 does have *blaZ*, the first alternative appears as a plausible explanation. While MRSA clinical strains carry type IV SCCmec, the dairy isolate IPLA3 has type V SCCmec. Type IV and V are relatively new types that replaced the previously prevalent types I and II. Interestingly, strain 132 has plasmid pUB110 (McKenzie et al., 1986; Gennimata et al., 1996) inserted into SCCmec type IVc that harbours kanamycin/tobramycin and bleomycin resistance genes as observed in previous isolates (Manara et al., 2018; Wang et al., 2021). Resistance to aminoglycosides was also frequent, generally due to the presence of *ant(4’)-Ia*, *aph(3’)Ia* and *aph(6)-Ic (str)* genes. In strains lacking these genes, adaptive resistance may be the reason for the resistant phenotypes, as described in previous studies (Chandrakanth et al., 2008). Some clinical strains also carry mutations that confer resistance to quinolones, but none of the food or mastitis isolates do.

The spread of virulence and antibiotic resistance genes can be greatly facilitated by mobile genetic elements. In this study, all strains had at least one pathogenicity island in their genomes, with IPLA13 being the strain with the highest number (13 SaPIs). However, these *S. aureus* pathogenicity islands (SaPIs) require a helper prophage for their mobilization (Novick and Ram, 2017). For instance, Dearborn and Dokland (2012) demonstrated that of the 4 prophages present in *S. aureus* Newman, wNM1 and wNM2 can mobilize SaPI1 and SaPIbov1. In this regard, most strains harbour intact temperate phages in their genomes. In addition to the aforementioned function, temperate phages might contribute to resistance gene transfer by transduction, and/or alter the expression of virulence factors in the host bacterium (Jurado et al., 2022). It is also worth mentioning the potential presence of plasmids in all but 7 strains of this study.

In addition to the prevalence of virulence and antibiotic resistance determinants, we also explored some phenotypes of the different strains, namely biofilm formation, staphyloxanthin biosynthesis, hemolysin production, proteolytic activity and colony spreading. Only three of the tested strains are strong biofilm formers, all of which were reference strains. These structures increase the persistence of *S. aureus* on surfaces, materials and food, complicating their elimination and treatment (Avila-Novoa et al., 2018). The mastitis strain V329 carries the biofilm-associated gene, *bap*, which allows a non-dependent form of polysaccharide intercellular adhesin (PIA) production for the formation of resistant cell aggregates (Lasa and Penadés, 2006). This gene is not present in any of the food or mastitis isolates from our collection. On the other hand, the strain 132 mutation in *agrC* could explain its high biofilm formation, given that the agr system participates in biofilm dispersion (Yarwood and Schlievert, 2003; Le and Otto, 2015). We should also mention the presence of genes involved in biofilm attachment (*clfA, clfB, atl, ebp, efb, fnbA, eap-map*) in most of the strains in our collection, regardless of their origin. However, it is also noteworthy that approximately 50% of the strains lack an intact *icaD* gene, which might explain their weak biofilm development. The strains were also quite homogeneous regarding other phenotypes. For instance, most strains produce staphyloxanthin, a pigment with antioxidant properties that defends the microorganism from the host immune system, and display both haemolytic and proteolytic activity. Fernandes et al. (2022) also demonstrated haemolytic activity in *S. aureus* strains from food handlers. However, this same study also found that 14.4% of the strains exhibited proteolytic activity, far from the percentage found in our collection of food strains (71%). In the food industry sector, this activity represents an important adaptation to the environment (Shaw et al., 2004), allowing the strains to use milk as a substrate (Anderson, 1976). Another study carried out with a CA-MRSA mutant strain demonstrated the importance of proteases for growth in peptide-rich environments, serum, in the presence of antimicrobial peptides (AMPs), and in human blood (Kolar et al., 2013).

Although *S. aureus* is regarded as a non-motile microorganism, we now know that cells can passively expand across the surface of soft agar plates through the production of surfactants (PSMs), a phenomenon called sliding or colony spreading (Pollitt et al., 2015). Apart for this function, PSMs exert many others related to pathogenesis (lysis of leukocytes and erythrocytes, stimulation of inflammatory responses, contribute to biofilm development), increasing the virulence of *S. aureus* strains (Gordon et al., 2014). Depending on their length, PSMs can be classified into alpha-type (20 to 25 amino acids) and beta-type (43 to 45 amino acids), with α PSMs conferring greater virulence (Wang et al., 2007). In our collection, all strains showed β-type PSMs, but only the clinical strains carried the alpha-type PSMs. This would explain why all strains in the study showed some degree of spreading. However, it is noteworthy that strains Sa9 and IPLA19, both from milk of mastitic cows, showed a high spreading capacity. This may be due to a mutation in the *hld* gene present in both strains. This PSM is regulated by the quorum-sensing system, and its production has been linked to a partial inhibition of spreading (Omae et al., 2012; Gordon et al., 2014). Mutations of the *hld* gene were also found in strains from other studies (Karki et al., 2020; Merda et al., 2020; Naushad et al., 2020). Given the role of PSMs in virulence, it would be interesting to track the prevalence of *hld* truncation in strains involved in cattle and human infections.

## 5 Concluding remarks

Tracking the prevalence of antibiotic resistance and virulence genes and phenotypes in *S. aureus* strains is very valuable in order to predict the potential risk of such traits spreading. Most studies of this kind focus on clinical isolates due to their direct impact on human health. However, it would be useful to intensify the characterization of *S. aureus* strains isolated in the food environment, since food would make it easier for isolates with a high pathogenic potential to reach the wider community. Here, we have focused on food isolates from Northern Spain and our results highlight the genetic diversity of such *S. aureus* isolates, including those isolated from the same location or within a short period of time. Nonetheless, there is a clear correlation between genetic proximity and source of the isolate. Some of the genotypic and phenotypic trends that we found in isolates coming from the same environment are similar to those identified in other studies. Nonetheless, other characteristics, such as the prevalent types of enterotoxins in mastitis isolates, seem to be specific to this particular collection, at least considering the information available at the moment. Finally, another interesting finding was the relatively low frequency of toxin-encoding genes and antibiotic resistance determinants in the food strains compared to strains from mastitic cows and to reference strains of clinical origin. Overall, our results suggest that possession of genes related to virulence and antibiotic resistance might not confer an advantage to *S. aureus* in the food environment. Therefore, strains carrying these determinants in foods might likely come from infected humans or animals. Nonetheless, it is important to remain vigilant and perform studies that track the prevalence of these genes and their corresponding phenotypes in food industry isolates and not only in clinical or veterinary strains.

## Data availability statement

The datasets presented in this study can be found in online repositories. The names of the repository/repositories and accession number(s) can be found below: https://www.ncbi.nlm.nih.gov/genbank/, CP134618; https://www.ncbi.nlm.nih.gov/genbank/, CP134619; https://www.ncbi.nlm.nih.gov/genbank/, CP134617; https://www.ncbi.nlm.nih.gov/genbank/, CP134620; https://www.ncbi.nlm.nih.gov/genbank/, JAVRXU000000000; https://www.ncbi.nlm.nih.gov/genbank/, JAVRXT000000000; https://www.ncbi.nlm.nih.gov/genbank/, JAVRXS000000000; https://www.ncbi.nlm.nih.gov/genbank/, JAVRXR000000000; https://www.ncbi.nlm.nih.gov/genbank/, JAVRXQ000000000; https://www.ncbi.nlm.nih.gov/genbank/, JAVRXV000000000; https://www.ncbi.nlm.nih.gov/genbank/, JAVRXX000000000; https://www.ncbi.nlm.nih.gov/genbank/, JAVRXW000000000.

## Author contributions

AJ: Data curation, Formal analysis, Investigation, Methodology, Visualization, Writing – original draft. LF: Conceptualization, Data curation, Formal analysis, Methodology, Supervision, Visualization, Funding acquisition, Writing – review & editing. AR: Conceptualization, Funding acquisition, Writing – review & editing. PG: Conceptualization, Funding acquisition, Supervision, Writing – review & editing.
