# Supplementary material for: Prevalence of virulence- and antibiotic resistance-associated genotypes and phenotypes in Staphylococcus aureus strains from the food sector compared to clinical and cow mastitis isolates
Source: Front Cell Infect Microbiol. 2024 Jan 29;14:1327131. doi: 10.3389/fcimb.2024.1327131 (PMC10859521; doi:10.3389/fcimb.2024.1327131)
Supplement: Supplementary file 1 [file Table_1.docx]

**Table S1. Genome sequencing, assembly and annotation of *Staphylococcus aureus* isolates**

| **Feature** | **Sa9** | **Sa5** | **Sa7** | **IPLA19** | **IPLA1** | **IPLA3** | **IPLA5** | **IPLA11** | **IPLA13** | **IPLA15** | **IPLA16** |
| --- | --- | --- | --- | --- | --- | --- | --- | --- | --- | --- | --- |
| Number of reads | 1,413,702 | 1,055,927 | 1,592,969 | 1,391,183 | 638,463 | 773,222 | 1,067,195 | 1,298,847 | 955,149 | 751,080 | 646,809 |
| *Assembly statistics*  Number of contigs | 1 | 71 | 91 | 99 | 54 | 80 | 69 | 76 | 42 | 2 | 1 |
| Number of contigs >1000 bp | 1 | 24 | 18 | 25 | 16 | 17 | 24 | 18 | 10 | 2 | 1 |
| Largest contig size (bp) | 2,708,529 | 729,480 | 590,174 | 744,252 | 727,211 | 679,388 | 723,296 | 654,733 | 784,445 | 2,781,582 | 2,750,073 |
| Genome size (bp) | 2,708,529 | 2,759,023 | 2,712,701 | 2,735,157 | 2,755,626 | 2,776,021 | 2,800,617 | 2,716,158 | 2,747,464 | 2,805,322 | 2,750,073 |
| G+C content (%) | 32.80 | 32.73 | 32.73 | 32.70 | 32.73 | 32.81 | 32.70 | 32.75 | 32.77 | 32.87 | 32.86 |
| N50 (bp) | 2,708,529 | 326,142 | 244,209 | 240,600 | 491,283 | 424,038 | 227,986 | 318,430 | 616,645 | 2,781,582 | 2,750,073 |
| Number of Ns per 100 kbp | 0 | 0 | 0 | 0 | 0 | 0 | 0 | 0 | 0 | 0 | 0 |
| *Annotation features* |  |  |  |  |  |  |  |  |  |  |  |
| Number of genes (coding) | 2,526 | 2,559 | 2,489 | 2,599 | 2,560 | 2,557 | 2,615 | 2,504 | 2,556 | 2,565 | 2,511 |
| Number of tRNAs | 60 | 61 | 61 | 59 | 60 | 61 | 60 | 63 | 61 | 60 | 61 |
| Number of rRNAs | ? | ? | ? | ? | ? | ? | ? | ? | ? | ? | ? |

*The reference *Staphylococcus aureus* strains are not shown (JE2, SH1000, MW2, 132, RN4220, Newman, and V329)

**TABLE S2. Identification of virulence genes in *Staphylococcus aureus* isolates**

| **Clinical** | | | | | | **Mastitis** | | | | | **Dairy industry** | | | **Meat industry** | | | |  |  |  |
| --- | --- | --- | --- | --- | --- | --- | --- | --- | --- | --- | --- | --- | --- | --- | --- | --- | --- | --- | --- | --- |
| JE2 | MW2 | 132 | RN4220 | Newman | V329 | | Sa9 | Sa5 | Sa7 | IPLA19 | IPLA1 | IPLA3 | IPLA5 | IPLA11 | IPLA13 | IPLA15 | IPLA16 |  |  |  |
| Adherence | *atl* | Autolysin | + | + | + | + | + | + | | + | + | + | + | + | + | + | + | + | + | + |
|  | *bap* | Biofilm-associated protein | - | - | - | - | - | + | | - | - | - | - | - | - | - | - | - | - | - |
|  | *ebh* | Cell wall associated fibronectin binding protein | + | + | - | + | - | + | | - | - | + | - | + | + | + | + | + | + | + |
|  | *clfA* | Clumping factor A | + | + | - | + | + | - | | + | - | - | - | - | + | + | - | - | - | - |
|  | *clfB* | Clumping factor B | + | + | - | + | + | - | | + | - | - | + | + | - | + | - | - | - | - |
|  | *cna* | Collagen adhesion | - | + | - | - | - | - | | - | - | - | - | - | - | - | - | + | + | - |
|  | *ebp* | Elastin binding protein | + | + | + | + | + | + | | + | + | + | + | + | + | + | + | + | + | + |
|  | *eap/map* | Extracellular adherence protein/MHC analogous protein | + | + | + | + | + | + | | + | + | + | + | + | + | + | + | + | + | + |
|  | *efb* | Fibrinogen binding protein | + | + | + | + | + | + | | + | + | + | + | + | + | + | + | + | + | + |
|  | *fnbA* | Fibronectin binding proteins | + | + | + | + | + | + | | + | + | + | + | + | + | + | + | + | + | + |
|  | *fnbB* |  | + | + | + | + | + | + | | - | + | + | - | + | + | + | + | + | - | + |
|  | *icaA* | Intercellular adhesin | + | + | + | + | + | + | | + | + | + | + | + | + | + | + | + | + | + |
|  | *icaB* |  | + | + | + | + | + | + | | + | + | + | + | + | + | + | - | + | + | + |
|  | *icaC* |  | + | + | + | + | + | + | | + | + | + | + | + | + | + | + | + | + | + |
|  | *icaD* |  | + | + | - | - | - | - | | + | + | - | + | - | + | - | - | - | + | - |
|  | *icaR* |  | + | + | + | + | + | + | | + | + | + | + | + | + | + | + | + | + | + |
|  | *sdrC* | Ser-Asp rich fibrinogen-binding proteins | + | + | - | + | + | - | | + | + | + | + | + | + | + | + | + | + | + |
|  | *sdrD* |  | + | + | - | + | + | - | | - | + | + | - | + | + | + | + | + | + | - |
|  | *sdrE* |  | + | + | - | - | + | + | | + | + | + | + | + | + | - | + | + | + | - |
|  |  |  |  |  |  |  |  |  | |  |  |  |  |  |  |  |  |  |  |  |
|  | *sdrF* |  | - | - | - | - | - | - | | - | - | - | - | - | - | - | - | - | - | - |
|  | *sdrG* |  | - | - | - | - | - | - | | - | - | - | - | - | - | - | - | - | - | - |
|  | *sdrH* |  | - | - | - | - | - | - | | - | - | - | - | - | - | - | - | - | - | - |
|  | *spa* | Staphylococcal protein A | + | + | + | + | + | + | | + | + | + | + | + | + | + | + | + | + | + |
| Enzyme | *sspB* | Cysteine protease | + | + | + | + | + | + | | + | + | + | + | + | + | + | + | + | + | + |
|  | *sspC* |  | + | + | + | + | + | + | | + | + | + | + | + | + | + | + | + | + | + |
|  | *hysA* | Hyaluronate lyase | + | + | + | + | + | + | | + | + | + | + | + | + | + | + | + | + | + |
|  | *geh* | Lipase | + | + | + | + | + | + | | + | + | + | + | + | + | + | + | + | + | + |
|  | *lip* |  | + | + | + | + | + | + | | + | + | + | + | + | + | + | + | + | + | + |
|  | *sspA* | Serine V8 protease | + | + | + | + | + | + | | + | + | + | + | + | + | + | + | + | + | + |
|  | *splA* | Serine protease | + | + | + | + | + | - | | + | + | + | + | + | - | + | + | + | + | + |
|  | *splB* |  | + | + | + | + | + | - | | + | + | + | + | + | - | + | + | + | + | + |
|  | *splC* |  | + | + | + | + | + | - | | + | + | + | + | + | - | + | + | + | + | + |
|  | *splD* |  | + | - | - | + | + | - | | - | + | + | - | + | - | + | + | + | + | + |
|  | *splE* |  | + | - | + | + | + | - | | + | + | - | + | - | - | + | + | + | + | + |
|  | *splF* |  | + | + | - | + | + | - | | + | + | - | + | - | - | + | + | + | + | + |
|  | *coa* | Staphylocoagulase | + | + | + | + | + | + | | + | + | + | + | + | + | + | + | + | + | + |
|  | *sak* | Staphylokinase | + | + | + | - | + | - | | - | - | + | - | - | - | - | - | + | - | - |
|  | *nuc* | Thermonuclease | + | + | + | + | + | + | | + | + | + | + | + | + | + | + | + | + | + |
| Immune evasion | *adsA* | AdsA | + | + | + | + | + | + | | + | + | + | + | + | + | + | + | + | + | + |
|  | *chp* | CHIPS | + | - | - | - | + | - | | - | - | - | - | - | - | - | + | - | - | - |
|  | *Undetermined* | Capsule | + | + | + | + | + | + | | + | + | + | + | + | + | + | + | + | + | + |
|  | *scn* | SCIN | + | + | + | - | + | - | | - | + | + | - | + | + | - | + | + | - | + |
|  | *sbi* | Sbi | + | + | + | + | + | + | | + | + | + | + | + | + | + | + | + | + | + |
| Secretion system | *esaA* | Type VII secretion system | + | + | + | + | + | + | | + | + | + | + | + | + | + | + | + | + | + |
|  | *esaB* |  | + | + | - | - | + | + | | + | + | + | + | + | - | + | + | + | + | + |
|  | *esaD* |  | + | + | + | + | + | - | | + | + | + | + | + | - | + | - | - | + | - |
|  | *esaE* |  | + | + | + | + | + | - | | + | + | + | + | + | - | + | - | - | + | - |
|  | *esaG* |  | + | + | + | + | + | + | | + | + | + | + | + | + | + | + | + | + | + |
|  | *essA* |  | + | + | + | + | + | + | | - | + | + | - | + | + | + | + | + | + | + |
|  | *essB* |  | + | + | + | + | + | + | | + | + | + | + | + | + | + | + | + | + | + |
|  | *essC* |  | + | + | + | + | + | + | | + | + | + | + | + | + | + | + | + | + | + |
|  | *esxA* |  | + | + | + | + | + | + | | + | + | + | + | + | + | + | + | + | + | + |
|  | *esxB* |  | + | + | + | + | + | - | | + | + | + | + | + | - | + | - | - | + | - |
|  | *esxC* |  | + | + | + | + | + | - | | + | + | + | + | + | - | + | - | - | + | - |
|  | *esxD* |  | + | + | + | + | + | - | | + | + | + | + | + | - | + | - | - | + | - |
| Toxin | *hly/hla* | Alpha hemolysin | + | + | + | + | + | + | | + | + | + | + | + | + | + | + | + | + | + |
|  | *hlb* | Beta hemolysin | - | + | + | + | + | + | | + | + | + | + | + | + | + | + | + | + | + |
|  | *hld* | Delta hemolysin | + | + | + | + | + | + | | - | + | + | - | + | + | + | + | + | + | + |
|  | *sea* | Enterotoxin A | - | + | + | - | + | - | | - | - | - | - | - | - | - | - | + | - | + |
|  | *seb* | Enterotoxin B | - | - | - | - | - | - | | - | - | - | - | - | - | - | - | - | - | - |
|  | *sec* | Enterotoxin C | - | + | - | - | - | - | | + | - | - | + | - | - | - | - | + | - | + |
|  | *sed* | Enterotoxin D | - | - | - | - | - | - | | - | - | - | - | - | - | - | - | - | - | - |
|  | *see* | Enterotoxin E | - | - | - | - | - | - | | - | - | - | - | - | - | - | - | - | - | - |
|  | *seg* | Enterotoxin G | - | - | - | - | - | - | | + | - | + | + | - | - | - | - | - | - | - |
|  | *seh* | Enterotoxin H | - | + | - | - | - | - | | - | - | - | - | - | - | - | - | - | + | - |
|  | *sei* | Enterotoxin I | - | - | - | - | - | - | | - | - | - | - | - | - | - | - | - | - | - |
|  | *sej* | Enterotoxin J | - | - | - | - | - | - | | - | - | - | - | - | - | - | - | - | - | - |
|  | *yent1* | Enterotoxin Yent1 | - | - | - | - | - | - | | - | - | + | - | - | - | - | - | - | - | - |
|  | *yent2* | Enterotoxin Yent2 | - | - | - | - | - | - | | - | - | + | - | - | - | - | - | - | - | - |
|  | *selk* | Enterotoxin-like K | + | + | - | - | - | - | | + | - | + | + | - | - | - | - | - | - | - |
|  | *sell* | Enterotoxin-like L | - | + | - | - | - | - | | + | - | - | - | - | - | - | - | - | - | - |
|  | *selm* | Enterotoxin-like M | - | - | - | - | - | - | | - | - | + | + | - | - | - | - | - | - | - |
|  | *seln* | Enterotoxin-like N | - | - | - | - | - | - | | - | - | + | + | - | - | - | - | - | - | - |
|  | *selo* | Enterotoxin-like O | - | - | - | - | - | - | | + | - | + | + | - | - | - | - | - | - | - |
|  | *selp* | Enterotoxin-like P | - | - | - | - | - | - | | - | - | - | - | - | - | - | - | - | - | - |
|  | *selq* | Enterotoxin-like Q | + | + | - | - | - | - | | - | - | - | - | - | - | - | - | - | - | - |
|  | *selr* | Enterotoxin-like R | - | - | - | - | - | - | | - | - | - | - | - | - | - | - | - | - | - |
|  | *selu* | Enterotoxin-like U | - | - | - | - | - | - | | + | - | - | + | - | - | - | - | - | - | - |
|  | *eta* | Exfoliative toxin type A | - | - | - | - | - | - | | - | - | - | - | - | - | - | - | - | - | - |
|  | *etb* | Exfoliative toxin type B | - | - | - | - | - | - | | - | - | - | - | - | - | - | - | - | - | - |
|  | *etc* | Exfoliative toxin type C | - | - | - | - | - | - | | - | - | - | - | - | - | - | - | - | - | - |
|  | *etd* | Exfoliative toxin type D | - | - | - | - | - | - | | - | - | - | - | - | - | - | - | - | - | - |
|  | *set10* | Exotoxin | - | - | - | - | - | - | | - | - | - | - | - | - | - | - | - | - | - |
|  | *set11* |  | - | - | - | - | - | + | | - | - | + | - | - | - | - | - | + | - | + |
|  | *set12* |  | - | - | - | - | - | - | | - | - | - | - | - | - | - | - | + | - | + |
|  | *set13* |  | - | - | - | - | - | + | | - | - | + | - | - | - | - | - | - | - | - |
|  | *set14* |  | - | - | - | - | - | - | | - | - | - | - | - | - | - | - | - | - | - |
|  | *set15* |  | - | - | - | - | - | - | | - | - | + | - | - | - | - | - | - | - | - |
|  | *set16* |  | - | + | - | - | - | + | | + | + | - | + | + | - | + | + | + | + | + |
|  | *set17* |  | - | + | - | - | - | - | | - | - | - | - | - | - | - | - | - | + | - |
|  | *set18* |  | - | + | - | - | - | + | | + | + | + | + | + | - | + | - | + | + | + |
|  | *set19* |  | - | + | - | - | - | + | | - | + | - | - | + | - | + | + | - | + | - |
|  | *set1* |  | - | - | - | - | - | - | | - | - | - | - | - | + | - | - | - | - | - |
|  | *set20* |  | - | - | - | - | - | - | | + | - | - | + | - | - | - | - | - | - | - |
|  | *set21* |  | - | + | - | - | - | - | | - | - | - | - | - | - | - | - | - | - | - |
|  | *set22* |  | - | + | - | - | - | - | | + | + | - | + | + | - | + | + | - | + | - |
|  | *set23* |  | - | + | - | - | - | - | | + | - | - | + | - | - | - | - | - | + | - |
|  | *set24* |  | - | + | - | - | - | - | | + | - | - | + | - | - | - | + | + | + | + |
|  | *set25* |  | - | + | - | - | - | - | | + | + | - | + | + | - | + | + | - | + | - |
|  | *set26* |  | - | + | - | - | - | + | | + | + | - | + | + | + | + | + | - | + | - |
|  | *set2* |  | - | - | - | - | - | - | | - | - | - | - | - | + | - | - | - | - | - |
|  | *set30* |  | + | - | + | + | + | - | | - | - | - | - | - | - | - | - | - | - | - |
|  | *set31* |  | + | - | + | + | + | + | | - | - | - | - | - | - | - | - | - | - | - |
|  | *set32* |  | + | - | + | - | + | - | | - | - | - | - | - | - | - | - | - | - | - |
|  | *set33* |  | + | - | + | - | + | - | | - | - | - | - | - | - | - | - | + | - | + |
|  | *set34* |  | + | + | + | + | + | + | | - | + | + | - | + | - | + | + | + | + | + |
|  | *set35* |  | + | - | + | + | + | - | | - | - | - | - | - | - | - | + | - | - | - |
|  | *set36* |  | + | - | + | + | + | - | | - | - | - | - | - | - | - | - | - | - | - |
|  | *set37* |  | + | - | + | + | + | - | | - | + | + | - | + | - | + | + | - | - | - |
|  | *set38* |  | + | - | + | + | + | - | | - | + | - | - | + | - | + | - | - | - | - |
|  | *set39* |  | + | - | + | + | + | + | | - | - | + | - | - | - | - | - | + | - | + |
|  | *set3* |  | - | - | - | - | - | - | | - | - | - | - | - | + | - | - | - | - | - |
|  | *set40* |  | - | - | + | + | + | - | | - | - | - | - | - | - | - | - | + | - | + |
|  | *set4* |  | - | - | - | - | - | - | | - | - | - | - | - | + | - | - | - | - | - |
|  | *set5* |  | - | - | - | - | - | - | | - | - | - | - | - | + | - | - | - | - | - |
|  | *set6* |  | - | - | - | - | - | - | | - | - | + | - | - | + | - | - | - | - | - |
|  | *set7* |  | - | - | - | - | - | - | | + | + | + | + | + | + | + | + | + | - | + |
|  | *set8* |  | - | - | - | + | - | - | | - | - | - | - | - | - | - | + | - | - | - |
|  | *set9* |  | - | - | - | + | - | - | | - | - | - | - | - | - | - | - | - | - | - |
|  | *hlgA* | Gamma hemolysin | + | + | + | + | + | + | | + | + | + | + | + | + | + | + | + | + | + |
|  | *hlgB* |  | + | + | + | + | + | + | | + | + | + | + | + | + | + | + | + | + | + |
|  | *hlgC* |  | + | + | + | + | + | + | | + | + | + | + | + | + | + | + | + | + | + |
|  | *lukF0like* | Leukocidin M | - | - | - | - | - | - | | - | - | - | - | - | - | - | - | - | - | - |
|  | *lukM* |  | - | - | - | - | - | - | | + | - | - | + | - | - | - | - | - | - | - |
|  | *lukD* | Leukotoxin D | + | + | + | + | + | - | | + | + | + | + | + | - | + | + | + | + | + |
|  | *lukE* | Leukotoxin E | - | - | - | - | - | - | | - | - | - | - | - | - | - | - | - | - | - |
|  | *lukF0PV* | Panton-Valentine leukocidin | + | + | - | - | - | - | | - | - | - | - | - | - | - | - | - | - | - |
|  | *lukS0PV* |  | + | + | - | - | - | - | | - | - | - | - | - | - | - | - | - | - | - |
|  | *tsst* | Toxic shock syndrome toxin | - | - | - | - | - | - | | + | - | - | - | - | - | - | - | - | - | - |
| Phenol-soluble modulins | PSMα-1 | Phenol-soluble modulins α | + | + | - | + | + | - | | - | - | - | - | - | - | - | - | - | - | - |
|  | PSMα-2 |  | + | + | + | + | + | - | | - | - | - | - | - | - | - | - | - | - | - |
|  | PSMα-3 |  | + | + | + | - | + | - | | - | - | - | - | - | - | - | - | - | - | - |
|  | PSMα-4 |  | - | - | + | - | - | - | | - | - | - | - | - | - | - | - | - | - | - |
|  | PSMβ-1 | Phenol-soluble modulins β | + | + | + | + | + | + | | + | + | + | + | + | + | + | + | + | + | + |
|  | PSMβ-2 |  | + | + | + | + | + | + | | + | + | + | + | + | + | + | + | + | + | + |
|  | PSMβ-3 |  | - | - | - | - | - | - | | - | - | - | - | - | - | - | - | - | - | - |
|  | PSMβ-4 |  | - | - | - | - | - | - | | - | - | - | - | - | - | - | - | - | - | - |

The virulome of 17 *S. aureus* isolates from different origins (Clinical sector, Mastitis, Food industry) based on the presence (+) or absence (-) of 137 enterotoxin genes.

**Table S3. Virulence gene analysis of *Staphylococcus aureus* isolates from other studies**

| **Strain** | **Country** | **Origin** | **Classical** | | | | | **New** | | | | | | | | | | | | | | | **PSM** | **GenBank** | **Ref** |
| --- | --- | --- | --- | --- | --- | --- | --- | --- | --- | --- | --- | --- | --- | --- | --- | --- | --- | --- | --- | --- | --- | --- | --- | --- | --- |
|  |  |  | ***sea*** | ***seb*** | ***sec*** | ***sed*** | ***see*** | ***seg*** | ***seh*** | ***sei*** | ***sej*** | ***yent1*** | ***yent2*** | ***selk*** | ***sell*** | ***selm*** | ***seln*** | ***selo*** | ***selp*** | ***selq*** | ***selr*** | ***selu*** | ***hld*** |  |  |
| SAU 1379 | Canada | Mastitis | 0 | 0 | 0 | 0 | 0 | 0 | 0 | 0 | 0 | 0 | 0 | 0 | 0 | 0 | 0 | 0 | 0 | 0 | 0 | 0 | - | JABWXB000000000.1 | Naushad et al. |
| SAU 0023 | Canada | Mastitis | 0 | 0 | 0 | 0 | 0 | 1 | 0 | 0 | 0 | 0 | 0 | 1 | 0 | 0 | 1 | 0 | 0 | 0 | 0 | 1 | 0 | JABWVM000000000.1 | Naushad et al. |
| SAU 0122 | Canada | Mastitis | 0 | 0 | 0 | 0 | 0 | 0 | 0 | 0 | 0 | 0 | 0 | 0 | 0 | 0 | 0 | 0 | 0 | 0 | 0 | 0 | 1 | JABWVR000000000.1 | Naushad et al. |
| SAU 0427 | Canada | Mastitis | 0 | 0 | 0 | 0 | 0 | 0 | 0 | 0 | 0 | 0 | 0 | 0 | 0 | 0 | 0 | 0 | 0 | 0 | 0 | 0 | 1 | JABWVZ000000000.1 | Naushad et al. |
| SAU 0842 | Canada | Mastitis | 0 | 0 | 0 | 0 | 0 | 0 | 0 | 0 | 0 | 0 | 0 | 0 | 0 | 1 | 1 | 0 | 0 | 0 | 0 | 1 | 0 | JABWWK000000000.1 | Naushad et al. |
| SAU 1169 | Canada | Mastitis | 0 | 0 | 0 | 0 | 0 | 0 | 0 | 0 | 0 | 0 | 0 | 0 | 0 | 0 | 0 | 0 | 0 | 0 | 0 | 0 | 1 | JABWWS000000000.1 | Naushad et al. |
| SAU 1710 | Canada | Mastitis | 0 | 0 | 0 | 0 | 0 | 0 | 0 | 0 | 0 | 0 | 0 | 0 | 0 | 0 | 0 | 0 | 0 | 0 | 0 | 0 | 1 | JABWXG000000000.1 | Naushad et al. |
| SAU 2236 | Canada | Mastitis | 0 | 0 | 0 | 0 | 0 | 0 | 0 | 0 | 0 | 0 | 0 | 0 | 0 | 0 | 0 | 0 | 0 | 0 | 0 | 0 | 1 | JABWXS000000000.1 | Naushad et al. |
| SAU 9314 | Canada | Mastitis | 0 | 0 | 0 | 0 | 0 | 0 | 0 | 0 | 0 | 0 | 0 | 0 | 0 | 0 | 0 | 0 | 0 | 0 | 0 | 0 | 1 | JABWZV000000000.1 | Naushad et al. |
| SAU 4506 | Canada | Mastitis | 0 | 0 | 0 | 0 | 0 | 1 | 0 | 0 | 0 | 0 | 0 | 0 | 0 | 1 | 1 | 1 | 0 | 0 | 0 | 1 | 0 | JABWYS000000000.1 | Naushad et al. |
| SAU 2526 | Canada | Mastitis | 0 | 0 | 0 | 0 | 0 | 1 | 0 | 0 | 0 | 1 | 0 | 0 | 0 | 1 | 1 | 0 | 0 | 0 | 0 | 0 | 0 | JABWXV000000000.1 | Naushad et al. |
| SAU 1553 | Canada | Mastitis | 0 | 0 | 0 | 0 | 0 | 0 | 0 | 0 | 0 | 0 | 0 | 0 | 0 | 0 | 0 | 0 | 0 | 0 | 0 | 0 | 1 | JABWXF000000000.1 | Naushad et al. |
| 1807M | Russia | Mastitis | 0 | 0 | 0 | 0 | 0 | 0 | 0 | 0 | 0 | 0 | 0 | 0 | 0 | 0 | 0 | 0 | 0 | 0 | 0 | 0 | 1 | NZ_WIQD01000010.1 | Fursova et al. |
| 8 | Russia | Mastitis | 0 | 0 | 0 | 0 | 0 | 0 | 0 | 0 | 0 | 0 | 0 | 0 | 0 | 0 | 0 | 0 | 0 | 0 | 0 | 0 | 1 | NZ_WIPT01000010.1 | Fursova et al. |
| 1709 | Russia | Mastitis | 0 | 0 | 0 | 0 | 0 | 0 | 0 | 0 | 0 | 0 | 0 | 0 | 0 | 0 | 0 | 0 | 0 | 0 | 0 | 0 | 1 | NZ_WNKR01000001.1 | Fursova et al. |
| 724 | Russia | Mastitis | 0 | 0 | 0 | 0 | 0 | 0 | 0 | 0 | 0 | 0 | 0 | 0 | 0 | 0 | 0 | 0 | 0 | 0 | 0 | 0 | 1 | NZ_WIPV01000010.1 | Fursova et al. |
| 615 | Russia | Mastitis | 0 | 0 | 0 | 0 | 0 | 1 | 0 | 0 | 0 | 0 | 1 | 1 | 0 | 1 | 1 | 1 | 0 | 0 | 0 | 0 | 1 | NZ_WIPP01000010.1 | Fursova et al. |
| 1838 | Russia | Mastitis | 0 | 0 | 0 | 0 | 0 | 1 | 0 | 1 | 0 | 0 | 1 | 0 | 0 | 1 | 1 | 1 | 0 | 0 | 0 | 0 | 1 | NZ_WIPQ01000010.1 | Fursova et al. |
| K68 | India | Mastitis | 0 | 0 | 0 | 0 | 0 | 0 | 0 | 0 | 0 | 0 | 0 | 0 | 0 | 0 | 0 | 0 | 0 | 0 | 0 | 0 | 1 | JAHSUU010000001.1 | Sivakumar et al. |
| B7 | India | Mastitis | 0 | 0 | 0 | 0 | 0 | 1 | 0 | 0 | 0 | 0 | 1 | 1 | 0 | 1 | 1 | 1 | 0 | 1 | 0 | 0 | 1 | JAHNUT010000001.1 | Sivakumar et al. |
| K181 | India | Mastitis | 0 | 0 | 1 | 0 | 0 | 0 | 0 | 0 | 0 | 0 | 0 | 0 | 0 | 0 | 0 | 0 | 0 | 0 | 0 | 0 | 1 | JAHLZT010000001.1 | Sivakumar et al. |
| BP5 | India | Mastitis | 0 | 0 | 0 | 0 | 0 | 0 | 0 | 0 | 0 | 0 | 0 | 0 | 0 | 0 | 0 | 0 | 0 | 0 | 0 | 0 | 1 | JAHNUX010000001.1 | Sivakumar et al. |
| K124 | India | Mastitis | 0 | 0 | 0 | 0 | 0 | 0 | 0 | 0 | 0 | 0 | 0 | 0 | 0 | 0 | 0 | 0 | 0 | 0 | 0 | 0 | 1 | JAHLZI010000001.1 | Sivakumar et al. |
| BP20 | India | Mastitis | 0 | 0 | 1 | 0 | 0 | 1 | 0 | 0 | 0 | 0 | 1 | 1 | 0 | 1 | 1 | 1 | 0 | 1 | 0 | 0 | 1 | JAHNVC010000001.1 | Sivakumar et al. |
| K3.1 | India | Mastitis | 0 | 0 | 0 | 0 | 0 | 1 | 0 | 0 | 0 | 0 | 1 | 1 | 0 | 1 | 1 | 1 | 0 | 1 | 0 | 0 | 1 | JAHNVH010000001.1 | Sivakumar et al. |
| B104A | USA | Meat (beef) | 0 | 0 | 0 | 0 | 0 | 0 | 0 | 0 | 0 | 0 | 0 | 0 | 0 | 0 | 0 | 0 | 0 | 0 | 0 | 0 | 1 | NZ_CP042048.1 | Karki et al. |
| B2015A | USA | Meat (chicken liver) | 0 | 0 | 0 | 0 | 0 | 1 | 0 | 0 | 0 | 1 | 0 | 1 | 0 | 1 | 1 | 1 | 0 | 0 | 0 | 0 | 0 | NZ_CP042043.1 | Karki et al. |
| B9022D | USA | Meat (pork) | 0 | 0 | 1 | 0 | 0 | 1 | 0 | 0 | 0 | 0 | 1 | 1 | 0 | 1 | 1 | 1 | 0 | 1 | 0 | 0 | 1 | NZ_CP042081.1 | Karki et al. |
| B3017D | USA | Meat (chicken) | 0 | 0 | 0 | 0 | 0 | 1 | 0 | 0 | 0 | 1 | 1 | 1 | 0 | 1 | 1 | 1 | 0 | 0 | 0 | 0 | 1 | NZ_CP042157.1 | Karki et al. |
| B6055A | USA | Meat (turkey) | 0 | 0 | 0 | 0 | 0 | 0 | 0 | 0 | 0 | 0 | 0 | 0 | 0 | 0 | 0 | 0 | 0 | 0 | 0 | 0 | 1 | NZ_CP042110.1 | Karki et al. |
| B4059C | USA | Meat (chicken) | 0 | 0 | 0 | 0 | 0 | 1 | 0 | 0 | 0 | 1 | 1 | 1 | 0 | 1 | 1 | 1 | 0 | 0 | 0 | 0 | 1 | NZ_CP042153.1 | Karki et al. |
| J028 | China | Meat (beef) | 1 | 0 | 1 | 0 | 0 | 1 | 0 | 0 | 0 | 1 | 1 | 1 | 1 | 1 | 1 | 1 | 0 | 0 | 0 | 0 | 1 | NZ_JAMRCV010000001.1 | Li et al. |
| J083 | China | Meat (chicken) | 1 | 0 | 1 | 0 | 0 | 1 | 0 | 0 | 0 | 1 | 1 | 1 | 1 | 1 | 1 | 1 | 0 | 0 | 0 | 0 | 1 | NZ_JAMRCV010000001.1 | Li et al. |
| J018 | China | Meat (pork) | 0 | 0 | 0 | 0 | 0 | 0 | 0 | 0 | 0 | 0 | 0 | 0 | 0 | 0 | 0 | 0 | 0 | 0 | 0 | 0 | 1 | NZ_JAMRBY010000001.1 | Li et al. |
| J033 | China | Meat (beef) | 0 | 0 | 0 | 0 | 0 | 0 | 0 | 0 | 0 | 0 | 0 | 0 | 0 | 0 | 0 | 0 | 0 | 0 | 0 | 0 | 1 | NZ_JAMRBU010000001.1 | Li et al. |
| J042 | China | Meat (chicken) | 0 | 0 | 0 | 0 | 0 | 0 | 0 | 0 | 0 | 0 | 0 | 0 | 0 | 0 | 0 | 0 | 0 | 0 | 0 | 0 | 1 | NZ_JAMQYQ010000001.1 | Li et al. |
| J050 | China | Meat (pork) | 0 | 1 | 0 | 0 | 0 | 0 | 0 | 0 | 0 | 0 | 0 | 0 | 0 | 0 | 0 | 0 | 0 | 0 | 0 | 0 | 1 | NZ_JAMRBT010000001.1 | Li et al. |
| P23 | Canada | Dairy | 1 | 0 | 0 | 0 | 0 | 0 | 0 | 0 | 0 | 0 | 0 | 0 | 0 | 0 | 0 | 0 | 0 | 0 | 0 | 0 | 1 | NZ_JAMZMR010000009.1 | Ashraf et al. |
| P5 | Canada | Dairy | 0 | 0 | 0 | 0 | 0 | 0 | 0 | 0 | 0 | 0 | 0 | 0 | 0 | 0 | 0 | 0 | 0 | 0 | 0 | 0 | 1 | NZ_JAMZMO010000009.1 | Ashraf et al. |
| P4 | Canada | Dairy | 0 | 0 | 0 | 0 | 0 | 0 | 0 | 0 | 0 | 0 | 0 | 0 | 0 | 0 | 0 | 0 | 0 | 0 | 0 | 0 | 1 | NZ_JAMZMN010000009.1 | Ashraf et al. |
| P18 | Canada | Dairy | 0 | 0 | 0 | 0 | 0 | 0 | 0 | 0 | 0 | 0 | 0 | 0 | 0 | 0 | 0 | 0 | 0 | 0 | 0 | 0 | 1 | NZ_JAMZMQ010000009.1 | Ashraf et al. |
| P7 | Canada | Dairy | 0 | 0 | 0 | 0 | 0 | 0 | 0 | 0 | 0 | 0 | 0 | 0 | 0 | 0 | 0 | 0 | 0 | 0 | 0 | 0 | 1 | NZ_JAMZMP010000009.1 | Ashraf et al. |
| 17SBCL533STA | France | Dairy | 0 | 0 | 0 | 0 | 0 | 0 | 1 | 0 | 0 | 0 | 0 | 0 | 0 | 0 | 0 | 0 | 0 | 0 | 0 | 0 | 1 | DANBNS010000001.1 | Merda et al. |
| 07CEB132STA | France | Dairy | 1 | 0 | 0 | 0 | 0 | 1 | 0 | 0 | 0 | 0 | 0 | 0 | 0 | 0 | 0 | 0 | 0 | 0 | 0 | 0 | 1 | DANBXO010000001.1 | Merda et al. |
| 11CEB272STA | Italy | Dairy | 1 | 0 | 0 | 0 | 0 | 1 | 0 | 0 | 0 | 0 | 0 | 0 | 0 | 0 | 0 | 0 | 0 | 0 | 0 | 0 | 1 | DANBWQ010000001.1 | Merda et al. |
| 11CEB274STA | Italy | Dairy | 0 | 0 | 1 | 0 | 0 | 0 | 0 | 0 | 0 | 0 | 0 | 0 | 0 | 0 | 0 | 0 | 0 | 0 | 0 | 0 | 1 | DANBWN010000001.1 | Merda et al. |
| 13CEB182STA | Ireland | Dairy | 0 | 0 | 1 | 0 | 0 | 1 | 0 | 0 | 0 | 0 | 0 | 1 | 1 | 1 | 1 | 1 | 0 | 0 | 0 | 1 | 0 | DANBIM010000001.1 | Merda et al. |
| 13CEB188STA | Ireland | Dairy | 1 | 0 | 0 | 0 | 0 | 1 | 0 | 0 | 0 | 1 | 1 | 1 | 0 | 1 | 1 | 1 | 0 | 0 | 0 | 0 | 1 | DANBPS010000001.1 | Merda et al. |
| 15SBCL1251STA | Algeria | Dairy | 1 | 1 | 0 | 0 | 0 | 0 | 0 | 0 | 0 | 0 | 0 | 0 | 0 | 0 | 0 | 0 | 0 | 0 | 0 | 0 | 1 | DANBOR010000001.1 | Merda et al. |
| 15SBCL1262STA | Algeria | Dairy | 1 | 1 | 0 | 0 | 0 | 0 | 0 | 0 | 0 | 0 | 0 | 0 | 0 | 0 | 0 | 0 | 0 | 0 | 0 | 0 | 1 | DANBON010000001.1 | Merda et al. |
| SA_SG_73 | Italy | Clinical | 0 | 0 | 0 | 0 | 0 | 1 | 0 | 0 | 0 | 0 | 1 | 1 | 0 | 1 | 1 | 1 | 0 | 0 | 0 | 0 | 0 | JAKNVL010000010.1 | Postiglione et al. |
| SA_SG_78 | Italy | Clinical | 0 | 0 | 0 | 0 | 0 | 0 | 0 | 0 | 0 | 0 | 0 | 0 | 0 | 0 | 0 | 0 | 0 | 0 | 0 | 0 | 0 | JAKJXR010000010.1 | Postiglione et al. |
| SA_SG_80 | Italy | Clinical | 0 | 0 | 0 | 0 | 0 | 1 | 0 | 1 | 0 | 0 | 1 | 0 | 0 | 1 | 1 | 1 | 0 | 0 | 0 | 0 | 0 | JAKNVH010000010.1 | Postiglione et al. |
| SA_SG_100 | Italy | Clinical | 1 | 0 | 0 | 0 | 0 | 1 | 0 | 0 | 0 | 0 | 0 | 1 | 0 | 1 | 1 | 1 | 0 | 0 | 0 | 1 | 1 | NZ_JAKNXI010000010.1 | Postiglione et al. |
| SA_SG_124 | Italy | Clinical | 0 | 1 | 1 | 0 | 0 | 1 | 1 | 0 | 0 | 0 | 0 | 0 | 0 | 0 | 0 | 1 | 0 | 0 | 0 | 1 | 0 | JAKNYD010000095.1 | Postiglione et al. |
| SA_SG_3 | Italy | Clinical | 1 | 0 | 0 | 0 | 0 | 1 | 0 | 0 | 0 | 1 | 1 | 1 | 0 | 1 | 1 | 1 | 0 | 0 | 0 | 0 | 1 | NZ_JAKJZD010000010.1 | Postiglione et al. |
| SA07B | UK | Clinical | 0 | 0 | 0 | 0 | 0 | 1 | 0 | 0 | 0 | 0 | 1 | 1 | 0 | 1 | 1 | 1 | 0 | 0 | 0 | 0 | 1 | CAMRFH010000001.1 | Mossop et al. |
| SA010B | UK | Clinical | 1 | 0 | 0 | 0 | 0 | 0 | 1 | 0 | 0 | 0 | 0 | 1 | 0 | 0 | 0 | 0 | 0 | 1 | 0 | 0 | 1 | CAMRFK010000001.1 | Mossop et al. |
| SA018 | UK | Clinical | 1 | 0 | 0 | 0 | 0 | 0 | 1 | 0 | 0 | 0 | 0 | 1 | 0 | 0 | 0 | 0 | 0 | 1 | 0 | 0 | 1 | CAMRFL010000001.1 | Mossop et al. |
| SA08 | UK | Clinical | 0 | 0 | 0 | 0 | 0 | 1 | 0 | 0 | 0 | 1 | 0 | 1 | 0 | 1 | 1 | 1 | 0 | 0 | 0 | 0 | 1 | CAMRFQ010000001.1 | Mossop et al. |
| SA012 | UK | Clinical | 1 | 0 | 0 | 0 | 0 | 1 | 0 | 0 | 0 | 1 | 1 | 1 | 0 | 1 | 1 | 1 | 0 | 0 | 0 | 0 | 1 | CAMRFV010000001.1 | Mossop et al. |
| SA016 | UK | Clinical | 0 | 0 | 0 | 0 | 0 | 1 | 1 | 0 | 0 | 0 | 0 | 1 | 0 | 1 | 1 | 1 | 0 | 0 | 0 | 1 | 1 | CAMRGD010000001.1 | Mossop et al. |
| 06b38 | China | Clinical | 0 | 1 | 0 | 0 | 0 | 0 | 0 | 0 | 0 | 0 | 0 | 1 | 0 | 0 | 0 | 0 | 0 | 1 | 0 | 0 | 1 | DAKZBR010000001.1 | Wang et al. |
| F35 | China | Clinical | 0 | 1 | 0 | 0 | 0 | 0 | 0 | 0 | 0 | 0 | 0 | 1 | 0 | 0 | 0 | 0 | 0 | 1 | 0 | 0 | 1 | DAKZBW010000001.1 | Wang et al. |
| W3254 | China | Clinical | 0 | 1 | 0 | 0 | 0 | 0 | 0 | 0 | 0 | 0 | 0 | 1 | 0 | 0 | 0 | 0 | 0 | 1 | 0 | 0 | 1 | DAKYUW010000001.1 | Wang et al. |
| R06 | China | Clinical | 1 | 1 | 0 | 0 | 0 | 0 | 0 | 0 | 0 | 0 | 0 | 1 | 0 | 0 | 0 | 0 | 0 | 1 | 0 | 0 | 1 | DAKYUE010000001.1 | Wang et al. |
| C09 | China | Clinical | 0 | 0 | 0 | 0 | 0 | 0 | 0 | 0 | 0 | 0 | 0 | 0 | 0 | 0 | 0 | 0 | 0 | 0 | 0 | 0 | 1 | DAKYUB010000001.1 | Wang et al. |
| lc050 | China | Clinical | 0 | 0 | 0 | 0 | 0 | 0 | 0 | 0 | 0 | 0 | 0 | 0 | 0 | 0 | 0 | 0 | 0 | 0 | 0 | 0 | 1 | DAKYXE010000001.1 | Wang et al. |
| S2300902017 | Australia | Clinical | 0 | 0 | 0 | 0 | 0 | 0 | 0 | 0 | 0 | 0 | 0 | 0 | 0 | 0 | 0 | 0 | 0 | 0 | 0 | 0 | 1 | DAOUNU010000001.1 | Coombs et al. |
| S6371602019 | Australia | Clinical | 0 | 0 | 0 | 0 | 0 | 0 | 0 | 0 | 0 | 0 | 0 | 0 | 0 | 0 | 0 | 0 | 0 | 0 | 0 | 0 | 1 | DAOUNS010000001.1 | Coombs et al. |
| S6253102020 | Australia | Clinical | 0 | 0 | 0 | 0 | 0 | 0 | 0 | 0 | 0 | 0 | 0 | 0 | 0 | 0 | 0 | 0 | 0 | 0 | 0 | 0 | 1 | DAOUNQ010000001.1 | Coombs et al. |
| 194 | Germany | Clinical | 0 | 0 | 1 | 0 | 0 | 1 | 0 | 0 | 0 | 0 | 1 | 1 | 1 | 1 | 1 | 1 | 0 | 0 | 0 | 0 | 1 | NZ_CP077932.1 | Wang, Hülpüsch et al. |
| 276 | Germany | Clinical | 0 | 0 | 0 | 0 | 0 | 0 | 0 | 0 | 0 | 0 | 0 | 0 | 0 | 0 | 0 | 0 | 0 | 0 | 0 | 0 | 1 | NZ_CP077921.1 | Wang, Hülpüsch et al. |
| 355 | Germany | Clinical | 1 | 0 | 0 | 0 | 0 | 0 | 0 | 0 | 0 | 0 | 0 | 0 | 0 | 0 | 0 | 0 | 0 | 0 | 0 | 0 | 1 | NZ_CP077885.1 | Wang, Hülpüsch et al. |
| AC4 | Germany | Clinical | 1 | 0 | 0 | 0 | 0 | 1 | 0 | 0 | 0 | 1 | 0 | 1 | 0 | 1 | 1 | 1 | 0 | 0 | 0 | 0 | 1 | NZ_CP077860.1 | Wang, Hülpüsch et al. |

The virulome of 75 *Staphylococcus aureus* isolates from different origins: 25 clinical, 25 food (dairy products, meat), 25 mastitis based on the presence (1) or absence (0) of 20 enterotoxin genes (Classical and New) and the state (1: intact, 0: no intact, -: absent) of a phenol-soluble modulin gene (δ-Haemolysin: *hld*). The selected studies belong to different geographical location (Germany, Australia, China, UK, Italy, Algeria, Ireland, France, Canada, Ireland, USA, India, and Russia)

References:

Ashraf, S., Naushad, S., Si, W., Bilal, M., Ijaz, M., Huang, H., and Zhao, X. (2022). Draft Genome Sequences and Antimicrobial Resistance Genes of Five *Staphylococcus aureus* Strains Isolated from Bovine Milk. *Microbiology resource announcements*, 11(10), e0075622. doi: 10.1128/mra.00756-22

Coombs, G. W., Daley, D., Shoby, P., Yee, N. W. T., Robinson, J. O., Murray, R., et al. (2022). Genomic characterisation of CC398 MRSA causing severe disease in Australia. *International journal of antimicrobial agents*, 59(4), 106577. doi: 10.1016/j.ijantimicag.2022.106577

Fursova, K., Sorokin, A., Sokolov, S., Dzhelyadin, T., Shulcheva, I., Shchannikova, M., et al. (2020). Virulence Factors and Phylogeny of *Staphylococcus aureus* Associated With Bovine Mastitis in Russia Based on Genome Sequences. *Frontiers in veterinary science*, 7, 135. doi:10.3389/fvets.2020.00135

Mossop, M., Robinson, L., Jiang, J. H., Peleg, A. Y., Blakeway, L. V., Macesic, N., et al. (2023). Characterisation of key genotypic and phenotypic traits of clinical cystic fibrosis *Staphylococcus aureus* isolates. *Journal of medical microbiology*, 72(6), 10.1099/jmm.0.001703. doi: 10.1099/jmm.0.001703

Postiglione, U., Batisti Biffignandi, G., Corbella, M., Merla, C., Olivieri, E., Petazzoni,. et al. (2023). Combining Genome Surveillance and Metadata to Characterize the Diversity of *Staphylococcus aureus* Circulating in an Italian Hospital over a 9-Year Period. *Microbiology spectrum*, 11(4), e0101023. doi: 10.1128/spectrum.01010-23

Sivakumar, R., Pranav, P.S., Annamanedi, M. et al. (2023). Genome sequencing and comparative genomic analysis of bovine mastitis-associated *Staphylococcus aureus* strains from India. *BMC Genomics* 24, 44. doi: 10.1186/s12864-022-09090-7
